# Integrative transcriptomic analysis and preclinical validation identify lansoprazole as a therapeutic candidate for preeclampsia

**DOI:** 10.1016/j.isci.2026.117963

**Published:** 2026-10-31

**Authors:** Romina Appierdo, Giang Pham, Ophelia Yin, Alice S. Tang, Tzu-yu Shao, Ashley L. Severance, Bahar Yilmaz, Jakob Einhaus, Ronald J. Wong, Francesco Vallania, Pier Federico Gherardini, Manuela Helmer-Citterich, Brice Gaudillière, Tomiko T. Oskotsky, David K. Stevenson, Sing Sing Way, Marina Sirota

**Affiliations:** 1 Bakar Computational Health Sciences Institute, University of California, San Francisco, San Francisco, CA, USA; 2 Center for Inflammation and Tolerance, Division of Infectious Diseases, Cincinnati Children’s Hospital Medical Center, Cincinnati, OH, USA; 3 Department of Pediatrics, Division of Neonatal and Developmental Medicine, Stanford University School of Medicine, Stanford, CA, USA; 4 Department of Biology, University of Rome Tor Vergata, Via della Ricerca Scientifica snc, 00133 Rome, Italy; 5 PhD Program in Cellular and Molecular Biology, Department of Biology, University of Rome “Tor Vergata”, Rome, Italy; 6 immunoLogic, San Francisco, CA, USA; 7 Machine Learning Collective, San Francisco, CA, USA

**Keywords:** drug repurposing, transcriptomics, pre-eclampsia

## Abstract

Preeclampsia is a pregnancy disorder with often severe consequences across the maternal-fetal dyad and limited treatment options. We performed an integrated meta-analysis of six placental transcriptomic datasets to identify a preeclampsia-specific gene expression signature, revealing dysregulated pathways related to hypoxia, immune dysfunction, and metabolism. Multivariate INTegrative (MINT) analysis was used to establish a smaller predictive signature with potential utility for identifying circulating biomarkers for earlier preeclampsia diagnosis and monitoring disease progression. This preeclampsia signature queried against a transcriptomic database of more than 1,000 compounds identified lansoprazole, a widely used proton pump inhibitor with an established safety profile, as the top-ranked compound based on its ability to reverse the preeclampsia-associated gene expression signature. Protective benefits of lansoprazole were confirmed using a murine model of immune-mediated fetal wastage where treatment overturned elevated soluble FLT1 levels with significantly improved pregnancy outcomes. These findings support further investigation of lansoprazole as a potential therapy for preeclampsia.

## Highlights

• Integrative transcriptomics identifies robust molecular signatures of preeclampsia
• Integrated analyses characterize molecular and cellular changes in preeclampsia
• Signature reversal identifies lansoprazole as a candidate for preeclampsia
• Lansoprazole improves pregnancy outcomes in a preclinical mouse model

## Graphical abstract

**Figure:**
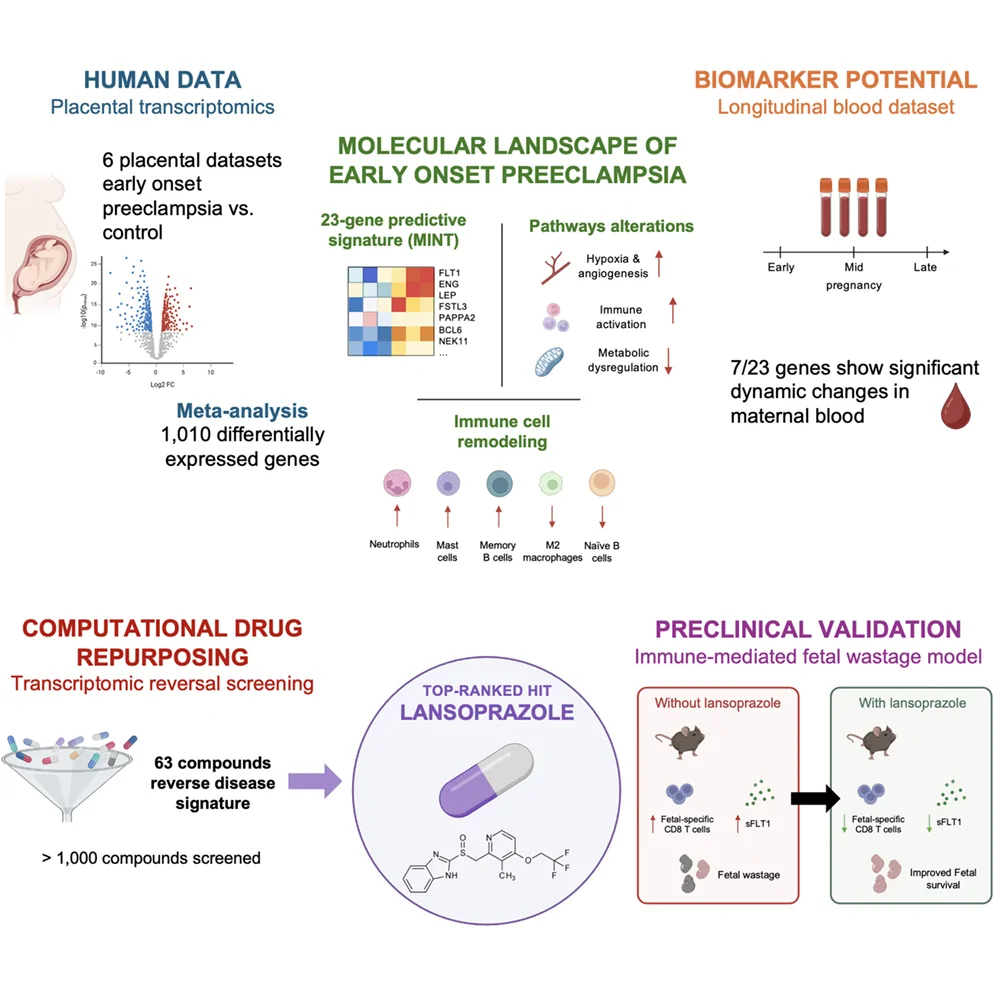

## Teaser

Pregnancy; Systems biology; Transcriptomics

## Introduction

Preeclampsia is an idiopathic inflammatory pregnancy disorder that affects ∼5% of pregnancies and is a major cause of perinatal morbidity and mortality.^1^^,^^2^ Preeclampsia typically develops after 20 weeks of gestation and is clinically characterized by new-onset hypertension, defined as a systolic blood pressure ≥140 mmHg and/or a diastolic blood pressure ≥90 mmHg measured on two occasions at least 4 h apart in a previously normotensive woman. The diagnosis is established in the presence of proteinuria (≥300 mg in a 24-h urine collection) and/or evidence of maternal end-organ dysfunction, including thrombocytopenia, renal insufficiency, impaired liver function, pulmonary edema, or neurological symptoms such as persistent headache and visual disturbances. Severe preeclampsia is characterized by blood pressure ≥160/110 mmHg and/or severe maternal complications including convulsions, hemolysis, hepatitis with elevated liver enzymes and low platelets.^3^ Women with preeclampsia have increased long-term risk of hypertension, cardiovascular disease, stroke, and metabolic disorders after pregnancy.^4^ In turn, children born from preeclamptic pregnancies are also at higher risk for complications such as preterm birth, low birth weight, and chronic conditions such as hypertension.^5^

The exact cause of preeclampsia is unknown, with complex and multifactorial etiologies implicated, often described as a two-stage model in which placental stress triggers a systemic maternal response.^6^ In the first stage, various pathological processes lead to placental dysfunction and syncytiotrophoblast stress at the maternal-fetal interface. In the second stage, the stressed placenta releases antiangiogenic and proinflammatory mediators into the maternal circulation, including soluble fms-like tyrosine kinase-1 (sFlt-1) and soluble endoglin (sEng), together with reduced placental growth factor (PlGF) availability. This angiogenic imbalance promotes systemic endothelial dysfunction, vascular inflammation, vasoconstriction, and increased vascular permeability, leading to the clinical manifestations of preeclampsia, including hypertension, proteinuria, and multiorgan dysfunction. Maternal conditions such as obesity, chronic hypertension, diabetes, and autoimmune diseases may further increase susceptibility to these placental signals and contribute to disease severity.^6^

Early-onset preeclampsia is defined as preeclampsia diagnosed before 34 weeks of gestation. Compared with late-onset disease, it is associated with greater maternal and fetal morbidity, including fetal growth restriction, preterm delivery, and increased risk of severe maternal complications, and is believed to arise predominantly from placental dysfunction and abnormal placentation. In early-onset preeclampsia, there is mainly defective trophoblast invasion and incomplete remodeling of the maternal spiral arteries, resulting in reduced placental perfusion, oxidative stress, and inflammation. Comparatively, late-onset preeclampsia may develop despite relatively normal placentation, likely because the increasing metabolic demands of the growing placenta exceed the maternal capacity to adequately support uteroplacental perfusion. Altered interactions between trophoblasts and maternal immune cells, including uterine NK cells, macrophages, and regulatory CD4 T (Treg) cells, further contribute to abnormal placental adaptation and inflammatory activation.

Unfortunately, our ability to identify at-risk pregnancies and effective therapies for preeclampsia remains rudimentary. While the most definitive treatment for preeclampsia remains delivery of the fetus and all products of conception, prophylactic low-dose aspirin, particularly when initiated early in gestation, has been shown to significantly reduce the incidence of preterm/early-onset preeclampsia in high-risk pregnancies.^7^ These considerations underscore urgent knowledge gaps regarding disease pathophysiology required for developing more targeted, disease-modifying therapies.^8^ The presented study is driven by substantial maternal and fetal morbidity and mortality associated with preeclampsia, particularly when diagnosed before 34 weeks of gestation, and accumulating evidence that early- and late-onset disease exhibit distinct molecular profiles.^9^^,^^10^^,^^11^ We first leveraged publicly available data in repositories such as the Gene Expression Omnibus (GEO)^12^ and ArrayExpress^13^ to investigate early-onset preeclampsia-specific molecular signatures and regulatory pathways. Furthermore, curated databases such as the Connectivity Map (CMap)^14^ and LINCS^15^ connect disease-associated gene expression signatures with molecular and genetic perturbations, creating a direct pathway from molecular characterization to therapeutic identification. We previously developed and validated a computational drug repurposing pipeline^16^ to identify candidate therapeutics with the hypothesis that strong therapeutic candidates will reverse the transcriptional profile of a disease signature back to a normal state. This pipeline has been successfully applied in prior studies to identify potential drug candidates for preterm birth^14^ and endometriosis,^15^ demonstrating potential ability to identify new, more effective treatments for complex diseases. Herein, this approach was used to identify lansoprazole as a potential therapy for early-onset preeclampsia, with therapeutic validation using an instructive preclinical model of fetal wastage triggered by partial depletion of maternal immune-suppressive FOXP3^+^ Treg cells. In this study we harnessed publicly available transcriptomics datasets to gain insights into the molecular underpinnings of preeclampsia and to identify therapeutic strategies through computational drug repurposing (Figure 1).

**Figure 1:**
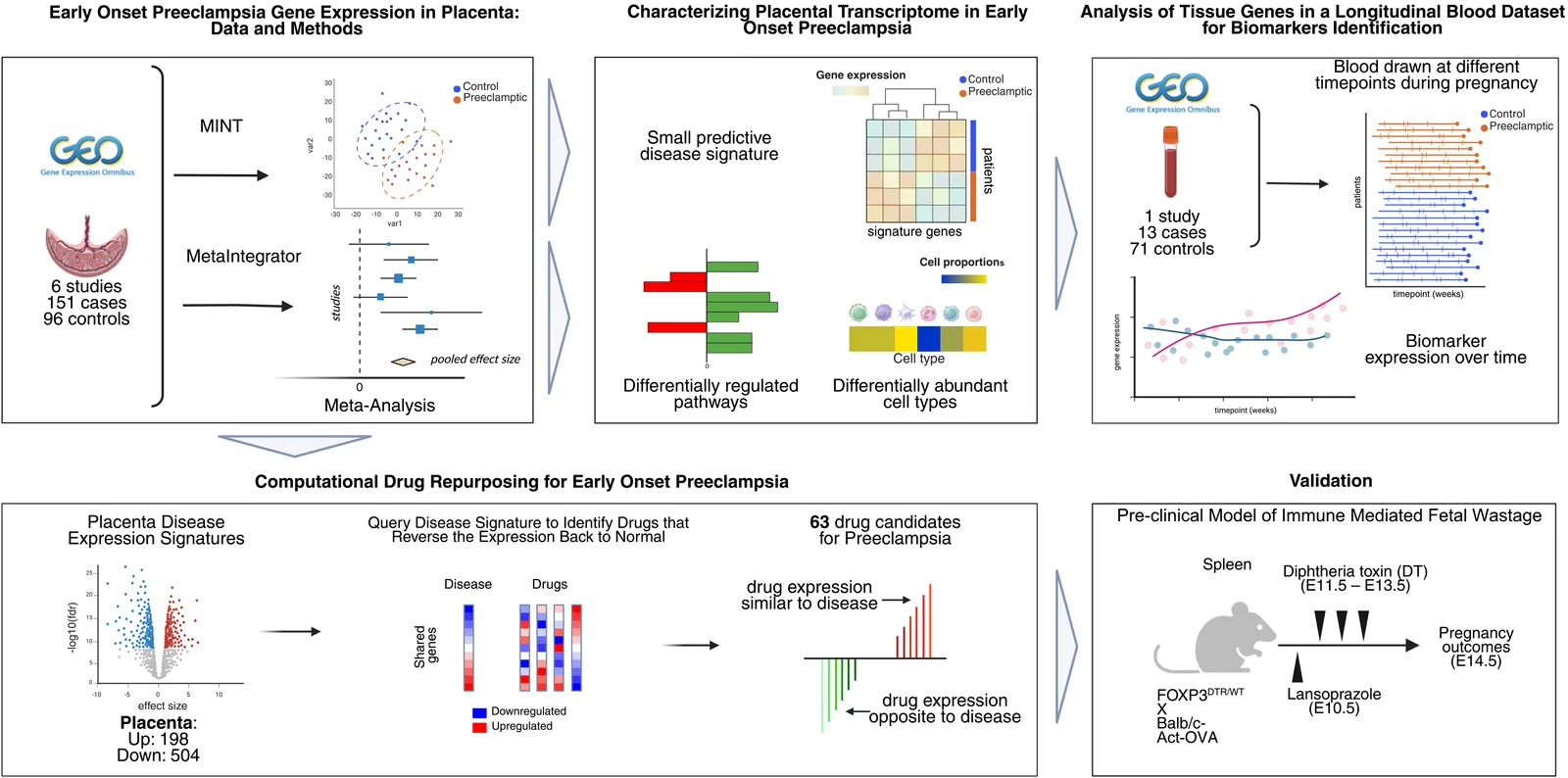
Study Overview: transcriptomics analysis leads to characterization of preeclampsia pathophysiology, biomarker identification, and candidate drug discovery We systematically collected six publicly available placental datasets from the Gene Expression Omnibus (GEO) to investigate the molecular underpinnings of preeclampsia. Meta-analysis was employed to define a large, comprehensive preeclampsia-specific disease signature (top-left image). We applied the MINT integration method to derive a concise predictive gene signature optimized for biomarker discovery (top middle image, above). Key genes from the MINT signature were then analyzed in blood to identify robust candidate biomarkers (top-right image). In parallel, meta-analysis was also used for pathway activity and cell type analyses to characterize the pathophysiology of the condition (top-middle image, below). The broader meta-analysis signature served as the foundation for the drug repurposing analysis, leading to the identification of 63 candidate therapeutics for preeclampsia (bottom-left image). Lansoprazole, the top-ranked candidate, was validated for efficacy in a preeclampsia mouse model, reinforcing its potential for future clinical applications (bottom-right image).

## Results

### Integrative transcriptomics analyses identifies potential early-onset preeclampsia biomarkers

We analyzed six placental transcriptomics datasets from the GEO database to identify robust biomarkers for early-onset preeclampsia. These datasets included a total of 151 samples from women with early-onset preeclampsia and 96 preterm controls (GA <37 weeks), ensuring GA-matched comparisons. The datasets were generated with multiple technologies, including both microarray (GSE25906,^16^ GSE74341,^17^ GSE75010,^18^ and GSE54618^19^) and RNA-seq platforms (GSE114691^20^ and GSE218039^21^). The distribution of samples across datasets ranged from 7 to 58 early-onset preeclampsia cases per study and 5 to 43 preterm controls (Table 1).

**Table 1:** Overview of the six placental transcriptomics datasets

Study	Sequencing technology	EO preeclampsia (ga ≤34 weeks)	Pre-term controls (ga <37 weeks)
GSE25906	microarray (GPL6102)	10	5
GSE74341	microarray (GPL16699)	7	5
GSE75010	microarray (GPL6244)	54	43
GSE54618	microarray (GPL10558)	7	7
GSE114691	RNA-seq (GPL11154)	58	21
GSE218039	RNA-seq (GPL24676)	15	15
		151	96

We comprehensively collected six publicly available placental datasets from GEO, profiling samples from women with or without a diagnosis of preeclampsia. For our analysis, we focused on women with early-onset (EO) preeclampsia, applying the canonical threshold of GA ≤34 weeks. As controls, we selected preterm samples (GA <37 weeks) to ensure appropriate matching for GA. This approach aimed to minimize confounding due to differences in pregnancy duration while isolating the molecular features of preeclampsia.

To integrate these datasets and minimize inter-study variability, we used the Multivariate INTegrative (MINT) method,^22^ implemented in the *mixOmics* R package.^23^ MINT integrates datasets while extracting common biological signals and minimizing cohort-specific biases. This approach allowed us to uncover a shared gene expression signature associated with early-onset preeclampsia across all cohorts. Before integration, dimensionality reduction of individual datasets revealed clear intra-study disease state clustering (Figure S1A). After integration, the MINT method aligned the datasets by extracting common components shared across all cohorts. In the resulting combined dimensionality reduction plot, samples were grouped by disease state rather than by study, indicating that the integration successfully uncovered shared transcriptomic patterns linked to EO preeclampsia (Figure S1B).

The MINT method identified a 23-gene signature (Figure 2A). This concise signature includes well-known players in preeclampsia and pregnancy-related processes, such as *FLT1*,^24^^,^^25^^,^^26^ leptin (*LEP*),^27^ *ENG*,^28^ follistatin-like 3 (*FSTL3*),^29^ and pregnancy-associated plasma protein A2 (*PAPPA2*).^30^ The enrichment of genes previously implicated in pregnancy-related processes such as preeclampsia provides additional validation of the robustness of the signature. Importantly, the remaining genes may represent novel biomarkers for preeclampsia, offering promising avenues for further exploration.

**Figure 2:**
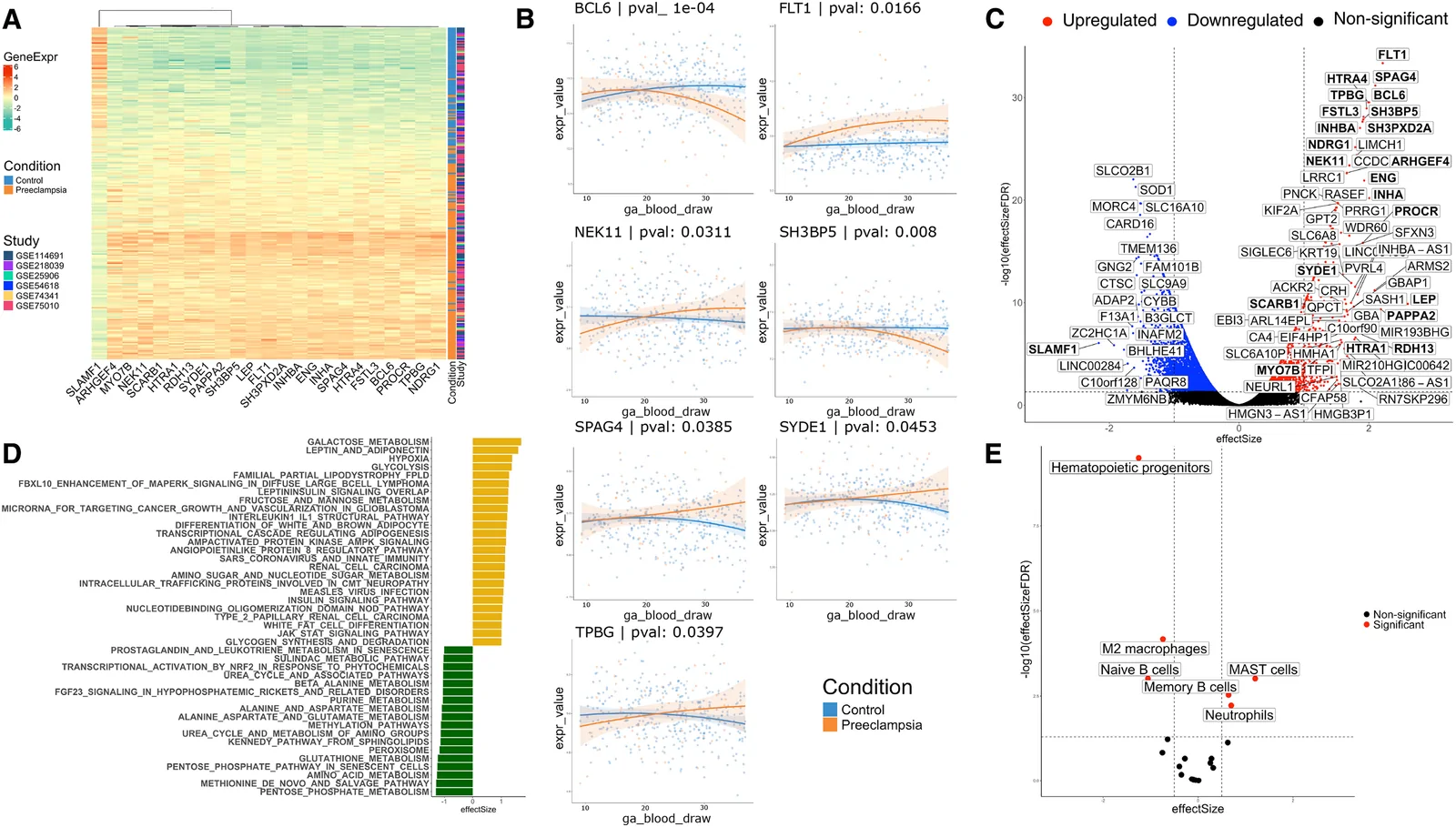
Cross-tissue transcriptomics analysis of early-onset preeclampsia identifies key pathways, cell types, and biomarkers Using the MINT method, we identified a concise, predictive gene signature for preeclampsia from the six placental datasets. This 23-gene signature includes several well-established biomarkers such as *FLT1*, along with *ENG*, *LEP*, and *PAPPA2*, all known to be implicated in preeclampsia or pregnancy-related processes. Additionally, less-studied genes such as *BCL6* and *NEK11* emerged as potential novel biomarkers (A). To assess the biomarker potential of the MINT signature, we analyzed a longitudinal blood dataset to evaluate gene expression trajectories over gestation in pregnancies affected by preeclampsia versus controls. Seven of the 23 MINT genes showed significant differences in longitudinal expression (*p* < 0.05). Longitudinal trajectories were analyzed using linear mixed-effects models including condition, gestational age modeled as a second-degree polynomial, and their interaction, with patient ID included as a random intercept. Lines represent model-predicted expression trajectories and shaded areas represent 95% confidence intervals. Displayed *p* values correspond to the interaction between condition and the linear component of gestational age. (B). We employed meta-analysis to define a larger, more comprehensive preeclampsia gene signature, integrating results from the six datasets. The volcano plot displays the final signature, which consists of 1,010 differentially expressed genes after filtering for FDR <0.05, significance in at least two datasets, and an absolute effect size >1 (C). Pathway analysis using ssGSEA combined with meta-analysis identified significantly deregulated pathways in preeclampsia. Pathway activity was quantified in each placental dataset using single-sample Gene Set Enrichment Analysis (ssGSEA) based on Hallmark, KEGG, and WikiPathways gene sets. ssGSEA scores were standardized prior to meta-analysis and integrated across the six placental datasets. The bar plot shows significantly dysregulated pathways in preeclampsia compared with controls, including upregulated hypoxia, leptin signaling, immune, and inflammatory pathways, and predominantly downregulated metabolic pathways (D). We performed cell-mixture deconvolution with meta-analysis to identify differentially abundant cell types in the placentas of preeclampsia cases versus controls. Immune cell populations, including neutrophils, M2 macrophages, and B cells, showed significant changes, highlighting their roles in the immune dysregulation and inflammatory polarization that characterize preeclampsia (E).

### Longitudinal sampling validates machine learning-based biomarker discovery for early-onset preeclampsia

To further assess the biomarker potential of the MINT signature, we analyzed the expression of these genes in blood samples to determine whether the placental signal could also be detected systemically. Given the scarcity of blood transcriptomics datasets in GEO that meet the criteria for integration analysis, our study leveraged one longitudinal dataset (GSE149437^31^). GSE149437, originally generated for preterm birth prediction studies, also contains samples from pregnancies complicated by early-onset preeclampsia and was therefore included in the present analysis, enabling us to maximize the value of existing datasets and gain additional insights into preeclampsia biology (Figure S2).

The dataset (GSE149437) included 13 patients with early-onset preeclampsia and 71 preterm controls (GA <37 weeks) (Table 2). In total, the analysis included 66 samples from early-onset preeclampsia cases and 355 samples from controls, with multiple time points sampled per patient, averaging ∼5 samples per case and ∼5 samples per control throughout pregnancy. The GAs for early-onset preeclampsia cases and controls were as follows: the mean GA at delivery was 31.69 weeks for cases and 34.1 weeks for controls (Wilcoxon test, *p* < 0.05) and the mean GAs at diagnosis were 31.13 and 33.74 weeks for cases and controls (Wilcoxon test, *p* < 0.05), respectively. Controls were diagnosed with either preterm premature rupture of membranes (PPROM) or spontaneous preterm delivery (sPTD). Blood samples were collected, on average, slightly earlier in cases (mean: 22.25 weeks) compared to controls (mean: 24.36 weeks) (Wilcoxon test, *p* < 0.05). The longitudinal design of the dataset provided a unique opportunity to identify potential dynamic changes in gene expression over time, capturing the progression of early-onset preeclampsia and its associated molecular signatures in the periphery.

**Table 2:** Overview of the blood longitudinal dataset

	EO preeclampsia	Pre-term controls
*n* patients	13	71
*n* samples	66	355

To enable cross-tissue analysis between placenta and blood, we collected a longitudinal blood dataset to support biomarker discovery, as blood is easier to collect and more practical for clinical use. We filtered the dataset to include 84 women: 13 with early-onset preeclampsia (EO) (defined as GA ≤34 weeks) and 71 preterm controls (defined as GA <37 weeks). Each participant was sampled multiple times during pregnancy, resulting in 66 samples from preeclamptic women and 355 from controls. This longitudinal dataset allowed us to track gene expression changes over time, offering valuable insights into the progression of preeclampsia and aiding in the identification of potential biomarkers.

To analyze the longitudinal gene expression data with repeated measures per patient, we used a linear mixed-effects model with fixed effects for time and disease state, and a random effect for patient ID to account for intra-patient correlation. Out of the 23 genes in the MINT-derived preeclampsia placental signature, seven showed statistically significant differences in expression over time between early-onset preeclamptic and control pregnancies in maternal peripheral blood. These findings underscore the dynamic nature of these genes and their potential as biomarkers for monitoring early-onset preeclampsia progression and diagnosis (Figure 2B).

Among the significant genes, *FLT1* re-emerged as a key player, serving as a positive control and further validating its critical role in preeclampsia. In addition to *FLT1*, two genes, B-cell lymphoma (*BCL6*) and never in mitosis gene A-related kinase 11 (*NEK11*), exhibited interesting trends in expression (Figure 2B). *BCL6* is a transcriptional repressor involved in the regulation of immune responses and inflammation,^32^ while *NEK11* is a kinase involved in DNA damage response and cell cycle regulation.^33^

### Larger meta-analysis signature reveals comprehensive insights into early-onset preeclampsia biology

While the MINT method was instrumental in identifying a concise biomarker signature for early-onset preeclampsia, we sought to expand our analysis to identify a more comprehensive set of differentially expressed genes (DEGs) associated with the disease. For this purpose, we employed the MetaIntegrator framework,^34^ a tool for performing meta-analysis of gene expression data. MetaIntegrator focuses on integrating datasets to identify DEGs by combining effect sizes across studies. This enables the detection of a broader disease signature by leveraging the collective strength of multiple datasets while mitigating the impact of cohort-specific biases.

We aggregated the six placental datasets, implementing standardized preprocessing steps to ensure consistency and reduce technical variability across studies (see Methods). The resulting meta-analysis identified a set of 1,010 DEGs (330 upregulated and 680 downregulated) (Table S1) in preeclampsia compared to controls, after filtering for statistical significance false discovery rate (FDR <0.05) and absolute effect size value (abs(ES) > 1) (Figure 2C). These genes, including FLT1 and BCL6, represented a comprehensive disease signature, capturing both well-known players in preeclampsia biology and novel candidates for further investigation. By complementing the targeted biomarker discovery of MINT with the hypothesis-free, large-scale integration of meta-analysis, we were able to gain a more holistic understanding of the transcriptomics landscape of preeclampsia. This integrative approach strengthens the foundation for identifying potential therapeutic targets and understanding disease mechanisms.

### Pathway analysis identifies hypoxia, *LEP* signaling, and immune dysregulation as central mechanisms in early-onset preeclampsia pathophysiology

To gain more interpretable insights into the molecular mechanisms of early-onset preeclampsia, we conducted a pathway activity analysis. For each sample in the six placental datasets, we employed the single-sample Gene Set Enrichment Analysis (GSEA) method.^35^^,^^36^ This approach allows the estimation of pathway activity for individual samples by calculating enrichment scores for predefined gene sets. Unlike traditional GSEA, which compares groups of samples, single-sample GSEA evaluates the relative expression ranks of genes within a single sample. It assigns higher scores to pathways where genes are consistently expressed at the top or bottom of the ranked list, providing a quantitative measure of pathway activity.

In order to get more biological insight into the identified signature, we utilized pathway collections from the Molecular Signatures Database (MSigDB),^37^ including curated gene sets (Hallmark pathways, Kyoto Encyclopedia of Genes and Genomes (KEGG) pathways, and WikiPathways), resulting in a total of 900 gene sets for the analysis. We performed a meta-analysis to identify pathways that were significantly differentially active between early-onset preeclampsia and control samples. Using thresholds of FDR <0.05 and abs(ES) > 1, we identified 43 differentially active pathways, with 25 upregulated and 18 downregulated (Figure 2D; Table S2). Hypoxia emerged as a key upregulated pathway, reflecting the activation of hypoxia-related molecular responses that have been previously reported in preeclampsia.^38^ Placental ischemia and impaired uteroplacental perfusion resulting from abnormal placentation are considered central features of disease pathogenesis, leading to ischemia-reperfusion injury, syncytiotrophoblast stress, the release of anti-angiogenic factors, and subsequent maternal systemic dysfunction.^39^ Another notable pathway is the leptin signaling, which is essential for energy homeostasis and placental function.^40^ Dysregulated *LEP* levels have been implicated in complications during pregnancy, including inflammation and vascular stress, both of which are prominent in preeclampsia.^41^ Additionally, several inflammatory and immune-related pathways were found upregulated, including *IL-1* signaling and innate immune signaling, highlighting the central role of immune dysregulation in its pathophysiology.^42^ Conversely, downregulated pathways were predominantly metabolic, including glycolysis/gluconeogenesis and fatty acid metabolism, suggesting disrupted energy metabolism in preeclampsia.^43^ These findings offer a comprehensive view of the biological processes underlying the condition and provide mechanistic insights into the complex interplay between placenta ischemia, immune activation and metabolic dysfunction. Such pathway-level analyses not only deepen our understanding of early-onset preeclampsia but also pave the way for hypothesis generation and identifying targeted interventions to address these dysregulated processes.

### Cell-mixture deconvolution identifies neutrophils, macrophages, and B cells in preeclampsia

Because immune dysregulation is increasingly recognized as a central component of preeclampsia pathogenesis, we sought to characterize immune cell composition across placental transcriptomic datasets. We employed cell-mixture deconvolution to analyze the six placental datasets, using the ImmunoStates basis matrix^44^ to estimate the proportions of 20 distinct immune cell types in our datasets (Figure 2E). This approach enabled us to systematically evaluate immune landscape alterations associated with preeclampsia across multiple cohorts and identify specific immune cell populations that may contribute to disease pathophysiology. The focus on immune cells is driven by their critical role in the pathophysiology of early-onset preeclampsia.^45^ Immune cells are key regulators of placental development and maternal-fetal immune tolerance, processes that are often dysregulated in preeclampsia.^45^ Understanding these immune alterations is essential for characterizing preeclampsia’s molecular mechanisms and identifying potential targets for therapeutic intervention.

To combine observations across datasets, we again used MetaIntegrator to perform a meta-analysis. For each cell type, we calculated a pooled effect size representing its differential abundance in early-onset preeclamptic compared to control placentas. Filtering by statistical significance (FDR <0.05 and abs(ES) > 0.5), we identified six cell types that showed differential abundance in disease: mast cells, neutrophils, and memory B cells were more abundant in early-onset preeclampsia, while hematopoietic progenitors, M2 macrophages, and naive B cells were less abundant (Figure 2E; Table S3). CD8^+^ T cells showed a slight, non-significant upregulation, with an effect size of 0.29. Neutrophils, macrophages, and both memory and naive B cells appear to be key drivers of immune dysregulation in preeclampsia. Dysregulated neutrophils might contribute to endothelial damage and oxidative stress,^42^ while a shift toward pro-inflammatory M1 macrophages could sustain inflammation and impair placental function.^42^ Similarly, an increase in memory B cells^42^ might disrupt immune tolerance, contributing to the inflammatory and vascular dysfunction characteristic of preeclampsia.

### Computational drug repurposing identifies lansoprazole as a potential therapeutic for early-onset preeclampsia

The identification of a robust early-onset preeclampsia gene signature provided an opportunity to systematically search for compounds capable of reversing disease-associated molecular perturbations. Transcriptomic reversal approaches are based on the premise that drugs inducing gene expression changes opposite to those observed in disease may help restore dysregulated biological pathways and improve disease-associated phenotypes. Therefore, we leveraged the Connectivity Map (CMap),^46^ a large-scale resource linking small molecules to their transcriptional effects, to identify candidate therapeutics predicted to reverse the molecular signature of early-onset preeclampsia. This approach enables unbiased prioritization of compounds based on systems-level molecular effects rather than preselected targets and has identified therapeutic candidates across a variety of complex diseases.^14^^,^^15^^,^^47^^,^^48^^,^^49^ Of the 1,010 genes in the preeclampsia signature, 702 overlapped with the Cmap (Table S6). This disease signature was subsequently utilized as input for our drug repurposing pipeline,^47^ which leverages a rank-based, nonparametric method to evaluate concordance between disease-associated gene expression profiles and drug-induced expression signatures in the CMap^46^ (Figure 3). The pipeline identified 63 drugs with significant potential to reverse the preeclampsia signature, defined by a *q*-value <0.05 and a negative reversal, or CMap, score (<0), indicating strong negative correlation with the disease signature (Figures 3A and 3B; Table S4). Among these candidates, several, such as irinotecan and camptothecin, have previously been proposed as potential therapies for preeclampsia in independent studies,^50^ demonstrating overlap between our findings and prior evidence. The top-ranked drug in our analysis was lansoprazole, a proton pump inhibitor widely used to manage gastric conditions, including acid reflux and peptic ulcers.^51^ Lansoprazole is an over-the-counter medication categorized as pregnancy category B, signifying a reassuring safety profile with no evidence of fetal harm.^52^^,^^53^

**Figure 3:**
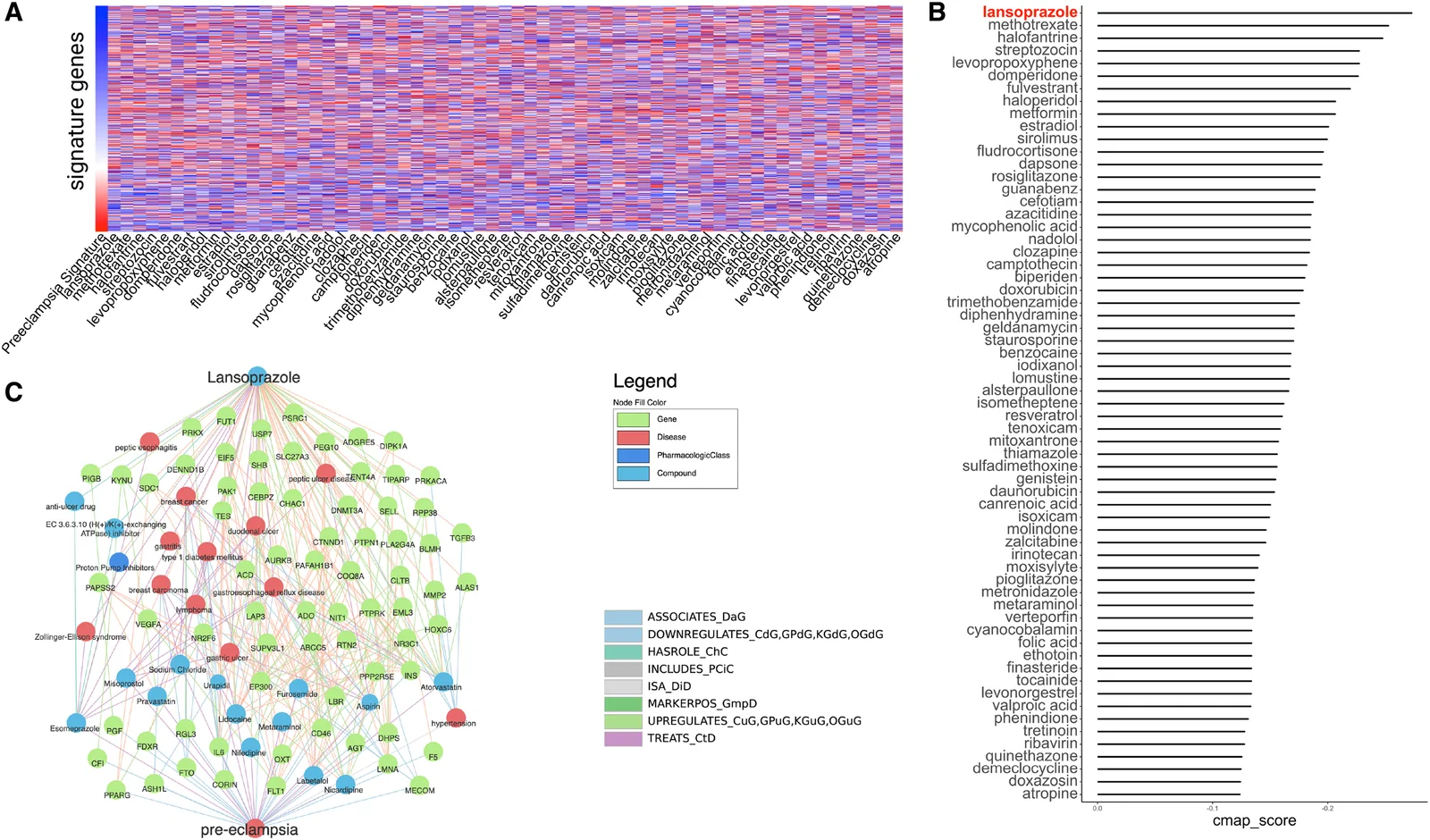
Computational drug repurposing identifies lansoprazole as the top candidate for preeclampsia treatment Using the meta-analysis gene signature as input, we employed a drug repurposing pipeline that utilizes a nonparametric rank-based method to assess the concordance between disease-associated gene expression profiles and drug-induced expression signatures. After filtering for statistical significance and CMap scores <0, we identified 63 drugs predicted to reverse the preeclampsia signature (A). Among these, the top-ranked candidate was lansoprazole, a widely available over-the-counter proton pump inhibitor classified as pregnancy category B, suggesting it is safe for use during pregnancy (B). SPOKE knowledge network reveals potential mechanistic links between lansoprazole and preeclampsia treatment. The network highlights relationships among compounds, genes, diseases, and pharmacologic classes using shortest paths. Lansoprazole is connected to preeclampsia through key gene nodes, including *FLT1*, *VEGFA*, and *PGF*. A specific pathway, “Lansoprazole –DOWNREGULATES→ *VEGFA* (Gene) ←(Gene Product)*UPREGULATES*(Gene)– *FLT1* (Gene) ←ASSOCIATES– pre-eclampsia”, illustrates a potential mechanism where *FLT1*, associated with preeclampsia, upregulates *VEGFA*, which is subsequently downregulated by lansoprazole. Node colors indicate genes (green), diseases (red), pharmacologic classes (blue), and compounds (dark blue), with edge types reflecting distinct biological and pharmacological relationships (C).

Leveraging prior literature and shortest-path analyses within the Scalable Precision Medicine Open Knowledge Engine (SPOKE) knowledge network,^54^ we identified relationships among genes, compounds, and disease-associated phenotypes that may provide biological context for the potential therapeutic effects of lansoprazole in preeclampsia (Figure 3C). Some specific gene nodes identified among the shortest paths include *FLT1*, *VEGFA*, and *PGF*. One specific path “Lansoprazole –DOWNREGULATES→ *VEGFA* (Gene) ←(Gene Product)_UPREGULATES_(Gene)– *FLT1* (Gene) ←ASSOCIATES– preeclampsia”.

Within the knowledge graph, lansoprazole was connected to preeclampsia through relationships involving angiogenesis-related genes, including FLT1, VEGFA, and PGF. However, these network-derived connections should be interpreted as hypothesis-generating associations rather than evidence of a direct biological mechanism. Notably, previous studies investigating the potential therapeutic role of lansoprazole in preeclampsia have reported mixed and sometimes contradictory results.^55^^,^^56^ While some studies have suggested potential effects on angiogenic pathways, others have failed to demonstrate consistent benefits. Therefore, the identification of lansoprazole in our analysis should be viewed as a computationally prioritized candidate that warrants further experimental and clinical investigation rather than confirmation of therapeutic efficacy.

### Sensitivity analyses confirm the robustness and specificity of molecular and therapeutic signals across disease presentations

As reported above, we utilized six independent placenta datasets to identify a disease signature specific to preeclampsia. Within each dataset, we selected cases of early-onset preeclampsia (defined as GA ≤34 weeks) as the disease group and preterm birth samples (GA <37 weeks) as controls, in order to match for GA. Using meta-analysis across these datasets, we identified a robust preeclampsia-associated gene signature that then we used to run the drug repurposing pipeline. However, because both preeclampsia and preterm birth are pregnancy complications and can share biological pathways—such as inflammation, altered placental development, and vascular dysfunction—we conducted a sensitivity analysis to ensure that the identified preeclampsia signature was specific to the disease and not confounded by preterm birth-related signals.

To perform this analysis, we utilized the same placenta datasets but focused on a different comparison: preterm birth controls (GA <37 weeks, 65 samples) versus term controls (GA ≥37 weeks, 75 samples). By applying meta-analysis to this comparison, we generated a preterm birth-specific gene signature that captured molecular features unique to preterm birth. After filtering for statistical significance (FDR <0.05), we identified a disease signature consisting of 784 differentially expressed genes, with 399 upregulated and 385 downregulated (Table S5). We then adjusted the original early-onset preeclampsia signature by subtracting the preterm birth signature, effectively isolating features uniquely associated with early-onset preeclampsia. This adjusted signature was then used as input for the drug repurposing pipeline described above. The results of the sensitivity analysis demonstrated that removing the preterm birth signal from the preeclampsia signature did not alter the outcomes of the drug repurposing pipeline. Specifically, nearly identical drug candidates were identified, and their rankings remained largely unchanged, even after accounting for potential confounding effects from preterm birth-related signals (Figure S3). This consistency suggested that the preeclampsia signature driving the drug repurposing analysis is robust and uniquely reflective of preeclampsia biology, rather than being influenced by overlapping or shared gene expression patterns with preterm birth.

To build on our initial findings, which centered on early-onset preeclampsia, we broadened the scope of our analysis to also include late-onset cases, carrying out a joint analysis that considered both early-onset and late-onset preeclampsia together. The datasets and number of patients collected from all available preeclampsia studies are listed in Table S1. We used all available controls and all available patients with preeclampsia, independently of the stage of delivery (term or preterm). In total, we included 181 controls and 225 preeclamptic patients. Gene expression meta-analysis identified a signature composed of 1,066 upregulated and 2,379 downregulated genes (FDR <0.05) (Figure S4). Genes such as FLT1, FSTL3, LEP, and ENG still came up from the analysis, consistent with what we expected for the disease.

Additionally, comparing either all genes or only significantly differentially expressed genes in both the early-onset preeclampsia vs. preterm birth analysis and the current combined analysis showed a strong positive correlation in effect sizes (Spearman ρ = 0.94, *p* < 0.05 for all genes; Spearman *ρ* = 0.94, *p* < 0.05 for significant genes) (Figures S4A–S4C). This indicates that the identified molecular signature is present in both early-onset and late-onset preeclampsia cases included in our analysis. In light of previous evidence demonstrating multiple placental transcriptomic subtypes of preeclampsia,^18^ these findings suggest that the signature may represent one molecular subtype that spans different gestational presentations rather than a universal feature of the disease.

We also performed a meta-analysis of pathway activity scores for the combined analysis, with leptin and hypoxia pathways emerging among the upregulated gene sets (Figure S5), further reinforcing the central role of placental metabolic stress and oxygen dysregulation in preeclampsia pathophysiology. Finally, we performed drug repurposing on the combined signature (Figure S6), which yielded 84 candidate drugs (q < 0.05 and CMap score <0), with lansoprazole still coming up as the top hit.

Sensitivity analyses demonstrated that the preeclampsia molecular signature and associated drug repurposing results, including identification of lansoprazole as the top candidate, were robust to potential confounding by preterm birth and remained highly consistent when both early- and late-onset preeclampsia cases were analyzed together.

### Lansoprazole attenuates the phenotype of preeclampsia in a murine immune-based model

Potential protective effects of lansoprazole were further evaluated using a preclinical model of fetal wastage triggered by partial depletion of maternal FOXP3^+^ Treg cells^57^ (Figure 4A). A hallmark feature of human pregnancy is expansion of CD4 T cells with immunosuppressive function identified by the FOXP3 transcription factor, called Treg cells.^58^^,^^59^^,^^60^^,^^61^^,^^62^^,^^63^ Pregnancy-induced expansion of maternal Treg cells has been proposed to sustain fetal tolerance, since inflammatory pregnancy complications such as preeclampsia are consistently associated with blunted expansion of maternal FOXP3^+^ CD4 T cells in the blood and decidual tissues.^58^^,^^59^^,^^60^^,^^61^^,^^62^^,^^63^ Similar expansion of maternal Treg cells occurs in mice, particularly during allogeneic pregnancy, whereas partial transient depletion of maternal FOXP3^+^ cells results in fetal wastage with reciprocal loss of live pups.^57^^,^^64^ This experimental platform using transgenic mice with co-expression of the high-affinity simian diphtheria toxin receptor (DTR) with FOXP3, exploiting the X-linked inheritance of *Foxp3* shared between mice and humans, and random inactivation of this chromosome in females, allowing for partial depletion of maternal Treg cells after diphtheria toxin (DT) administration to FOXP3^DTR/WT^ dams.^57^^,^^65^^,^^66^

**Figure 4:**
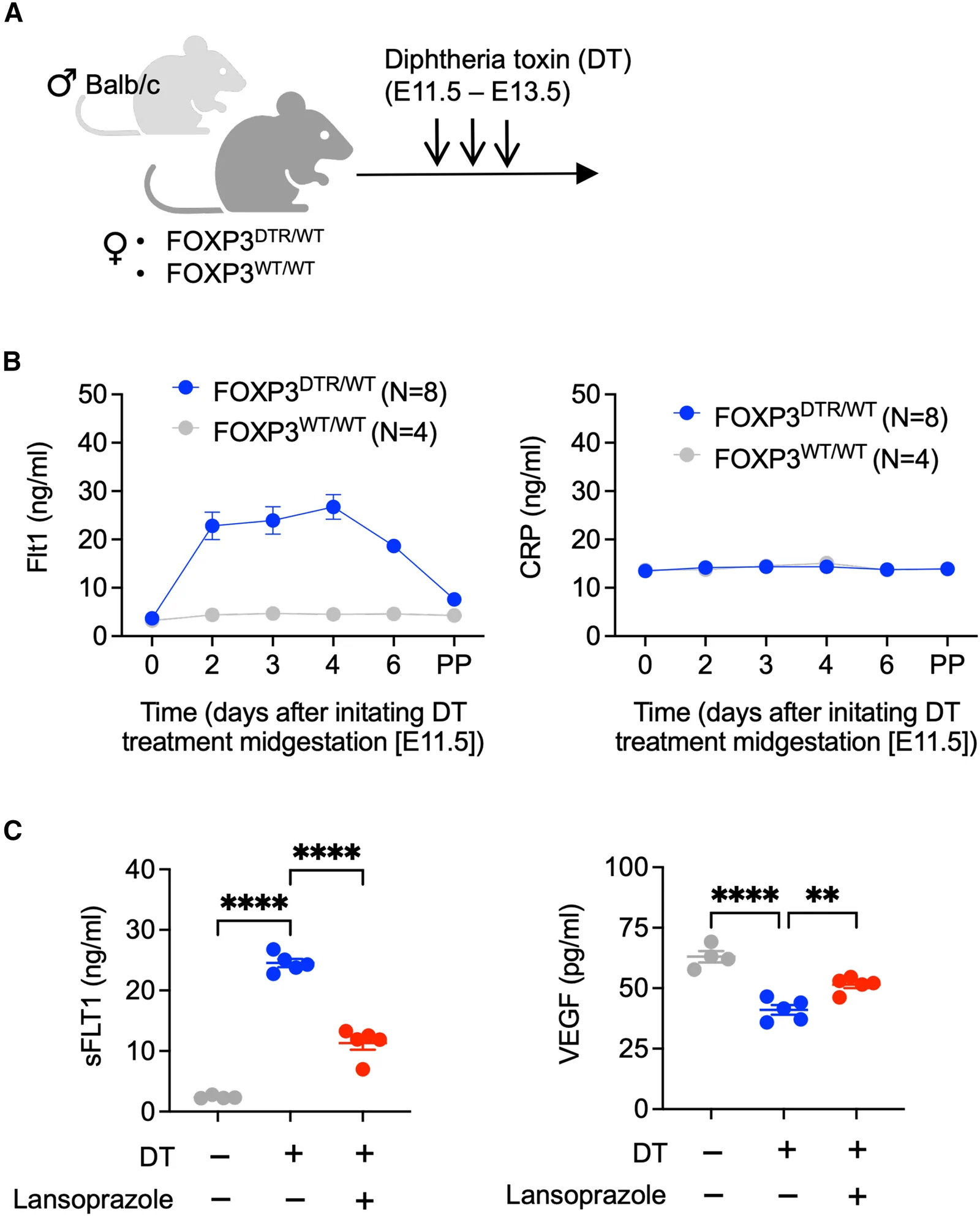
Lansoprazole overrides sFLT1 accumulation triggered by partial transient depletion of maternal FOXP3^+^ Treg cells (A) Breeding scheme and experimental schematic: FOXP3^DTR/WT^ or FOXP3^WT/WT^ female mice, each on the C57BL/6 background, during allogeneic pregnancy sired by males on the Balb/c background, are administered diphtheria toxin (DT) daily from embryonic day (E)11.5 to E13.5 to partially deplete maternal FOXP3^+^ Treg cells. (B) Serum sFLT1 and CRP levels at each time point after initiating DT treatment for each group of mice described in A. (C) Serum sFLT1 and VGEF levels in FOXP3^DTR/WT^ dams 24 h after initiating DT treatment with or without lansoprazole, compared with control mice without DT treatment. Ordinary one-way ANOVA; ∗∗*p* < 0.01; ∗∗∗∗*p* < 0.001. Error bars represent mean ± SEM.

Partial depletion of maternal Treg cells achieved by DT administration to FOXP3^DTR/WT^ midgestation pregnant mice causes sharply increased serum levels of sFLT1 similar to preeclampsia in humans and non-human primates^67^^,^^68^ (Figure 4B). Sustained background levels in DT-treated control FOXP3^WT/WT^ pregnant mice demonstrate that DT-induced sFLT1 in FOXP3^DTR/WT^ pregnant mice is specific to depletion of maternal Treg cells and is not explained by potential non-specific effects of DT (Figure 4B). Perturbations after partial depletion of maternal FOXP3^+^ Treg cells in this context causing fetal wastage also do not reflect non-specific inflammation, given only background serum C-reactive protein (CRP) levels in DT-treated FOXP3^DTR/WT^ and FOXP3^WT/WT^ mice (Figure 4B). Importantly, sFLT1 levels are sharply reduced with reciprocally increased VEGF in FOXP3^DTR/WT^ mice administered lansoprazole one day prior to initiating DT treatment (Figure 4B). Thus, partial depletion of maternal Treg cells to pre-pregnancy levels in mice, and blunted expansion of these cells in human preeclampsia share increased circulating sFLT1 as a common molecular signature which is efficiently overturned by lansoprazole. Increased sFLT1 after partial depletion of maternal FOXP3^+^ Treg cells in this context also dovetails nicely with the aforementioned analytical data highlighting differential expression of this VEGFR in human preeclampsia, and the necessity for sFLT1 in non-human primate preeclampsia models.^67^

Next, the impacts of lansoprazole were evaluated using male mice engineered to constitutively express a cell surface recombinant protein containing the model antigen OVA to sire allogeneic pregnancy in FOXP3^DTR/WT^ dams, thereby transforming OVA into a surrogate fetal antigen^57^^,^^69^ (Figure 5A). These experiments showed the expected ∼40% fetal wastage in FOXP3^DTR/WT^ pregnant mice after initiating DT treatment midgestation with reciprocal loss of live concepti (Figure 5B). Remarkably, these consequences associated with partial depletion of maternal Treg cells are attenuated in mice administered lansoprazole one day prior to DT administration (Figure 5B). Thus, lansoprazole protects against fetal wastage induced by partial depletion of maternal Treg cells.

**Figure 5:**
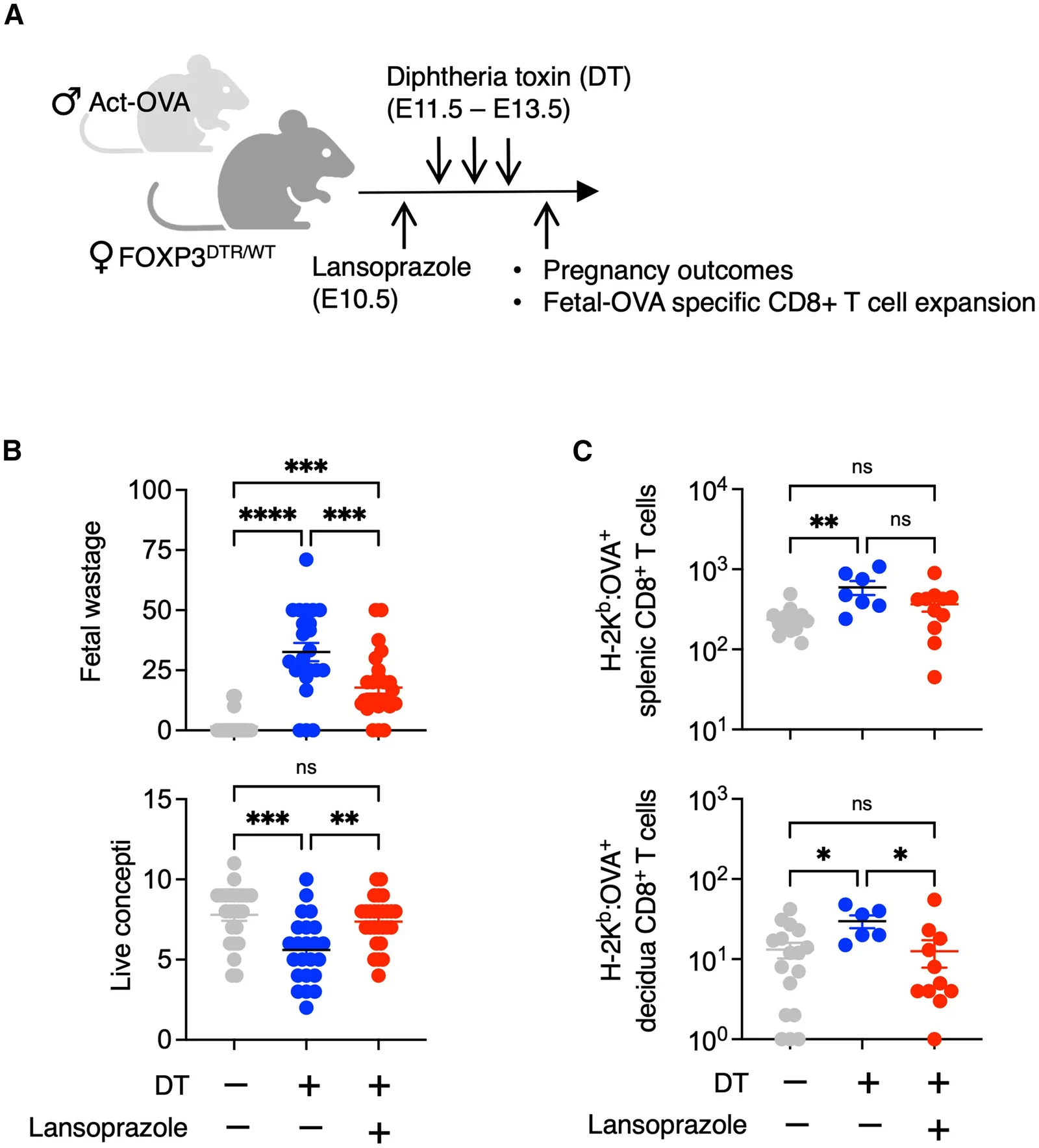
Lansoprazole protects against fetal wastage triggered by partial transient maternal FOXP3^+^ Treg cell depletion Breeding scheme and experimental schematic: allogeneic pregnancy in FOXP3^DTR/WT^ female mice on the C57BL/6 background, sired by Act-OVA males on the Balb/c background, transforming OVA into a surrogate fetal antigen (A). Percent fetal wastage and number of live concepti per litter for FOXP3^DTR/WT^ midgestation mice treated with DT alone or DT plus lansoprazole (B). CD8 T cells with fetal-OVA specificity identified by H-2K^b^:OVA_257-264_ tetramer staining among splenocytes (top) or decidual leukocytes (bottom) for FOXP3^DTR/WT^ midgestation mice treated with DT alone or DT plus lansoprazole (C). Ordinary one-way ANOVA; ns = not significant; ∗*p* < 0.05; ∗∗*p* < 0.01; ∗∗∗*p* < 0.005. Error bars represent mean ± SEM.

Fetal injury induced by maternal Treg cell depletion is associated with expansion and decidual infiltration of activated effector CD8 T cells with fetal specificity which drive fetal wastage.^70^^,^^71^ To investigate how lansoprazole impacts the dynamics and distribution of maternal effector T cells, CD8 T cells with fetal OVA-specificity were enumerated after staining with H2K^b^:OVA_257-264_ tetramers (74). These analyses showed expansion of fetal-OVA-specific CD8 T cells in the spleen and decidua in mice with fetal wastage induced by partial transient depletion of maternal FOXP3^+^ Treg cells, which was overturned in lansoprazole-treated mice (Figure 5C). The effects of lansoprazole were most pronounced in the decidua where fetal-OVA CD8 T cells were reduced to background levels found in control mice without DT treatment (Figure 5C). Thus, improved pregnancy outcomes in lansoprazole treated mice was associated with reduced systemic and decidual infiltration of fetal-specific maternal CD8 T cells unleashed by partial depletion of maternal FOXP3^+^ Treg cells which replicates the blunted expansion of these cells in human preeclampsia.^58^^,^^59^^,^^60^^,^^61^^,^^62^^,^^63^

## Discussion

This project leveraged public transcriptomics data to deepen our understanding of early-onset preeclampsia pathophysiology, including identification of disease-specific gene signatures and potential candidates for therapeutic intervention. Importantly, computational findings were validated through experimental studies, including a preclinical model of fetal wastage with vascular dysfunction shown by high sFLT1 levels to further establish translational relevance. We performed a cross-tissue analysis, focusing on six placental datasets profiling samples from both preeclamptic and control individuals. Using multiple datasets allowed us to mitigate study-specific biases, ensuring that our observations were robust, reliable, and broadly reproducible. Data integration was central to our approach, and therefore, we employed multiple methodologies, each tailored to the specific objectives of our analysis.

We first identified a concise 23-gene predictive signature for preeclampsia using the MINT algorithm. Notably, many of these genes are already recognized for their critical roles in preeclampsia and pregnancy-related biological processes, underscoring the robustness and biological relevance of the signature. Among the most prominent genes identified was *FLT1*, a well-characterized biomarker for preeclampsia, usually showing increasing expression over pregnancy.^72^ sFLT1 plays a key role in regulating angiogenesis, and its dysregulation is a hallmark of preeclampsia, reflecting the imbalance between pro- and anti-angiogenic factors that drive placental dysfunction.^25^^,^^72^ Other noteworthy genes included *ENG*, linked to vascular dysfunction, and *LEP, FSTL3,* and *PAPPA2*, which are involved in inflammation, placental development, and growth factor signaling, respectively.^73^^,^^74^^,^^75^^,^^76^ Interestingly, the MINT analysis identified both previously reported preeclampsia-associated genes, such as BCL6,^77^ and less extensively studied candidates, including NEK11.^78^ BCL6 is involved in the regulation of immune responses and inflammation; its increased expression in preeclampsia suggests a potential role in modulating the heightened immune activity and inflammation observed in the disease. NEK11 is instead involved in DNA damage response and cell cycle regulation. Dysregulation of NEK11 in preeclampsia may reflect cellular stress and impaired placental function, both of which are hallmark features of the condition. This demonstrates MINT’s ability to go beyond validating established biomarkers by identifying novel candidates, offering new opportunities for advancing research into the disease.

To broaden our analysis across tissues, we examined the expression of the identified genes in a peripheral blood dataset. Blood-based biomarkers offer a significant clinical advantage due to the ease and non-invasive nature of sample collection, enabling earlier diagnosis and potential intervention prior to delivery. Additionally, the dataset was longitudinal, capturing multiple time points during pregnancy. This longitudinal design provided crucial insights into the temporal dynamics of gene expression changes associated with preeclampsia, enhancing our understanding of progression over time. We leveraged the GSE149437 dataset,^31^ which profiled blood samples from both preeclamptic and control individuals at multiple time points during pregnancy. Focusing on the MINT-derived gene signature from our placental analysis, we utilized linear mixed-effects models to assess the behavior of these genes in the blood over time. Notably, seven of the 23 placental-derived genes exhibited significant differential expression and consistent directionality across pregnancy in the periphery, indicating robust differences between preeclampsia and control pregnancies. Among these, *FLT1* once again served as a positive control, reaffirming its established role in preeclampsia. Additionally, genes such as *NEK11* and *BCL6* emerged as intriguing candidates for further investigation. These genes, while not as widely studied in the context of preeclampsia, demonstrated significant and distinct expression patterns, suggesting their potential as biomarkers. Beyond their diagnostic performance, the identified blood-based biomarkers may have potential clinical utility for both early detection and disease monitoring in preeclampsia. In principle, such markers could be assessed during early pregnancy to identify women at increased risk of developing the disease and, therefore, candidates for preventive or therapeutic interventions. Furthermore, longitudinal monitoring of these biomarkers throughout gestation could provide insights into disease progression and treatment response. However, these potential applications remain speculative and will require validation in prospective cohorts with longitudinal sampling before clinical implementation.

While MINT is highly effective for identifying small, predictive signatures suitable for biomarker discovery, we also aimed to define a larger and more comprehensive preeclampsia gene signature by focusing on differentially expressed genes across studies. To accomplish this, we utilized meta-analysis, a statistical method that synthesizes results from multiple studies to calculate a pooled effect size. This approach allowed us to determine the overall differential expression of each gene by integrating data across the six placental datasets. This approach yielded a robust preeclampsia gene signature consisting of 1,010 differentially expressed genes, with 330 upregulated and 680 downregulated. Notably, all 23 genes from the MINT-derived signature were also present in the meta-analysis signature, underscoring the robustness and consistency of our methodologies. Additionally, our placental gene signature shows substantial concordance with the canonical preeclampsia transcriptome described over the past two decades, while also highlighting features that extend beyond it. The most strongly upregulated transcripts in our dataset—FLT1, LEP, INHBA, INHA, ENG, FSTL3, PAPPA2, SIGLEC6, and NDRG1-recapitulate the core “canonical” PE module first delineated by Sitras et al. in severe preeclamptic placentas^72^ and later formalized by Leavey et al. through unsupervised clustering of 330 placentas, in which FSTL3, FLT1, LEP, INHA, INHBA, PAPPA2 and ENG emerged as the top discriminators of the canonical/placental subtype.^18^^,^^79^ The convergent dysregulation of FLT1 and ENG in our data is consistent with the antiangiogenic axis (sFlt-1/sEng vs. PlGF) that underpins the leading mechanistic model of preeclampsia and that has been validated as a clinical predictor of impending disease,^80^ and the FLT1 signal aligns with the fetal-genome preeclampsia risk locus identified by the McGinnis et al.^81^ Concordance is further supported by the multi-omics synthesis of Than et al., who consolidated FLT1, ENG, LEP and PAPPA2 as a robust “blood-pressure gene module” detectable at both placental and circulating-protein levels.^82^ On the downregulated side, our finding of reduced SLCO2B1, SLC16A10, SLC9A9, SLC6A8, F13A1, CYBB, ADAP2 and SLAMF1 expression is consistent with the broader pattern of impaired placental solute-carrier transport and attenuated trophoblast/macrophage homeostatic transcripts described in previous bulk transcriptomic studies. Specifically, the meta-analysis and qPCR validation of Ajmeriya et al. confirmed downregulation of SLC6A8 and SLC16A10 in preeclamptic placentas across multiple GEO cohorts,^83^ the RNA-seq study of Sõber et al. reported decreased F13A1 and FAM101B in late-onset preeclampsia with intrauterine growth restriction,^84^ and the RNA-seq analysis of Kaartokallio et al. likewise highlighted dysregulation of immune and vascular transcripts in pre-eclamptic placentae.^85^

Our meta-analysis signature provided a broader view of the transcriptional landscape of preeclampsia and served as the foundation for the next stage of our research, namely the drug repurposing analysis. By leveraging this expanded gene set, we aimed to identify therapeutic candidates capable of targeting the molecular mechanisms implicated in the disease. We then sought to further characterize the pathophysiology of preeclampsia by employing featurization methods to derive higher-order, more interpretable features from the gene expression data. Specifically, we performed pathway activity and cell type analyses on the six preeclampsia datasets, offering a deeper biological context and insight into the underlying mechanisms.

For the pathway analysis, we computed pathway activity scores for each patient within each dataset, enabling us to quantify the activity of specific biological pathways. By applying meta-analysis to pool observations across datasets, we identified differentially active pathways associated with preeclampsia. Notably, the results highlighted the upregulation of pathways related to hypoxia and lipid metabolism. Placental ischemia resulting from abnormal placentation is a central feature of preeclampsia pathogenesis and is associated with the activation of hypoxia-responsive pathways, contributing to syncytiotrophoblast stress, the release of anti-angiogenic factors, and maternal systemic dysfunction.^86^ Similarly, dysregulation of lipid metabolism, particularly involving leptin signaling, emerged as a prominent feature of preeclampsia in our analysis, consistent with a substantial body of previous literature.^27^^,^^40^^,^^41^ LEP, a hormone critical for energy balance and placental function, has long been implicated in the pathophysiology of preeclampsia and other pregnancy-related complications, with numerous studies reporting altered leptin expression and signaling in affected pregnancies.^27^^,^^40^ Additionally, we observed upregulation in immune response pathways, underscoring the interplay between inflammation, immunity, and preeclampsia development.

To investigate the relationship between altered biological pathways and immune cell behavior in preeclampsia, we used cell mixture deconvolution to estimate cell-type proportions in placental samples. Meta-analysis of these estimates revealed significant shifts in immune cell populations, with neutrophils, macrophages, and B cells standing out as key players in the disease’s pathology.^45^ Neutrophils are particularly striking because of their complex roles in both healthy pregnancies and preeclampsia. Under normal conditions, neutrophils contribute to placental development by producing IL-8, a chemokine that supports angiogenesis, endothelial activation, and cell migration.^87^ However, in preeclampsia, these processes become dysregulated. Excessive neutrophil infiltration into vascular tissues leads to the release of oxidative stress molecules, exacerbating endothelial dysfunction. Beyond their structural roles, neutrophils also modulate the immune environment by regulating cytokine production and T cell activity.^42^ In preeclampsia, this immunoregulatory function appears disrupted, contributing to immune imbalance and amplifying systemic inflammation.

Macrophages also reflect the altered immune landscape in preeclampsia. During a healthy pregnancy, anti-inflammatory M2 macrophages dominate after the second trimester, supporting tissue repair, angiogenesis, and immune tolerance.^88^ This balance naturally shifts toward pro-inflammatory M1 macrophages during labor, but in preeclampsia, this shift occurs prematurely, sustaining chronic inflammation, impairing placental function, and intensifying systemic inflammatory responses.^42^ B cells play a similarly pivotal role in immune regulation during pregnancy. Normally, B cells foster a tolerant immune environment, often through interactions with Tregs. In preeclampsia, however, an increase in memory B cells undermines this balance. While Tregs retain their suppressive activity on B cell proliferation, the dysregulation of memory B cells fuels a pro-inflammatory environment that destabilizes immune tolerance and disrupts placental development.^42^ More broadly, these immune alterations should be interpreted within the emerging view that the maternal-fetal interface is organized into spatially distinct immune niches composed of trophoblasts, decidual stromal cells, uterine natural killer (uNK) cells, macrophages and T cell populations that collectively regulate placental development and immune tolerance. Recent spatial analyses have demonstrated that disruption of these immune microenvironments contributes to pregnancy disorders, including preeclampsia, highlighting the importance of considering both cellular composition and spatial organization when interpreting placental immune signatures.^89^

In addition to adaptive immune mechanisms, increasing evidence supports a central role for uNK cells in coordinating immune-vascular interactions during pregnancy. Through the secretion of cytokines, growth factors, and angiogenic mediators, uNK cells regulate trophoblast invasion, spiral artery remodeling, and maternal-fetal immune tolerance. Dysregulation of these pathways has been linked to impaired placental vascular development and the pathogenesis of preeclampsia.^90^ Therefore, the immune alterations observed in our study may reflect not only inflammatory dysregulation but also broader disturbances in immune-vascular crosstalk at the maternal-fetal interface.

Together, these findings reinforce the critical role of immune dysregulation in preeclampsia and provide further evidence of the complex interplay between immune activation, oxidative stress, inflammation and angiogenic imbalance. When considered alongside recent models of spatial placental immune organization and uNK-mediated immune-vascular regulation, our results support the concept that preeclampsia arises from coordinated disturbances across multiple immune and vascular pathways rather than from dysfunction of any single immune cell population.^89^^,^^90^ These analyses offered valuable insights into the molecular and cellular mechanisms underlying preeclampsia, providing a biologically rich context that informed the subsequent drug repurposing phase of the study.

The meta-analysis-derived preeclampsia gene signature was subsequently utilized in our drug repurposing pipeline to identify potential therapeutic candidates reversing the disease expression signature. After filtering for statistical significance and selecting drugs with a negative reversal score, we identified 63 candidates, with lansoprazole emerging as the top hit. Lansoprazole, a widely used over-the-counter proton pump inhibitor for gastric conditions, is classified as pregnancy category B, indicating a favorable safety profile for use during pregnancy.^52^ This classification is particularly important given the necessity of balancing efficacy and safety in therapeutic agents during pregnancy. While lansoprazole’s primary mechanism of action involves inhibition of the gastric proton pump (H^+^/K^+^ ATPase) to reduce gastric acid secretion,^91^ its potential benefits in preeclampsia suggest additional mechanisms may be involved, possibly related to its anti-inflammatory or vascular effects. Identification of lansoprazole as a leading candidate for preeclampsia treatment was especially compelling given our prior findings that this compound demonstrated efficacy in addressing preterm birth,^14^ another pregnancy-related condition.

To test lansoprazole’s efficacy in preeclampsia, we employed a mouse model designed to replicate critical features of the disease, including elevated sFLT1 levels, a hallmark of preeclampsia associated with anti-angiogenic imbalances and systemic dysfunction.^68^ This model was particularly valuable because it also mimicked the maternal immune adaptations that occur during pregnancy, with a focus on the role of immune suppressive Treg cells.^57^^,^^58^^,^^59^^,^^60^^,^^61^^,^^62^^,^^63^ Treg cells, identified by expression of FOXP3, are essential for maternal immune tolerance, preventing the rejection of the genetically foreign fetus by suppressing excessive immune activation.^57^^,^^58^^,^^59^^,^^60^^,^^61^^,^^62^^,^^63^^,^^64^^,^^65^ Blunted expansion or diminished suppressive function of maternal Treg cells is strongly linked to pregnancy complications such as preeclampsia and spontaneous abortion.^57^^,^^58^^,^^59^^,^^60^^,^^61^^,^^62^^,^^63^^,^^64^ In this model, we specifically investigated the consequences of partial depletion of maternal FOXP3^+^ Treg cells to mimic immune dysfunction observed in preeclampsia. This was achieved by using FOXP3^DTR^ transgenic mice engineered to co-express the simian high-affinity diphtheria toxin receptor (DTR) with FOXP3, allowing experimental manipulation of Treg cells based on their lineage-defining marker.^58^^,^^59^ DT administration to pregnant FOXP3^DTR/WT^ dams allows for synchronized and controlled depletion of maternal Tregs to pre-pregnancy levels.^57^^,^^66^ This depletion resulted in a phenotype characterized by fetal wastage with reciprocally reduced numbers of live pups. Mechanistically, fetal loss in this context was associated with heightened activation of maternal CD8 T cells and infiltration of these cells into the decidua, which targeted fetal antigens in a manner analogous to graft rejection in organ transplantation.^68^^,^^92^ Accumulation of these activated cytotoxic T cells underscores the importance of Treg cells in maintaining immune balance and protecting the fetus. Importantly, recapitulating both immune dysregulation and elevated sFLT1 levels in this model creates a robust platform for testing lansoprazole, which significantly mitigated the consequences of maternal Treg cell depletion. The drug not only reduced the accumulation of activated CD8 T cells in the decidua and systemic secondary lymphoid tissues, but also restored and efficiently protected against immune-mediated fetal wastage. The proposed disease mechanism and therapeutic model is summarized in Figure 6.

**Figure 6:**
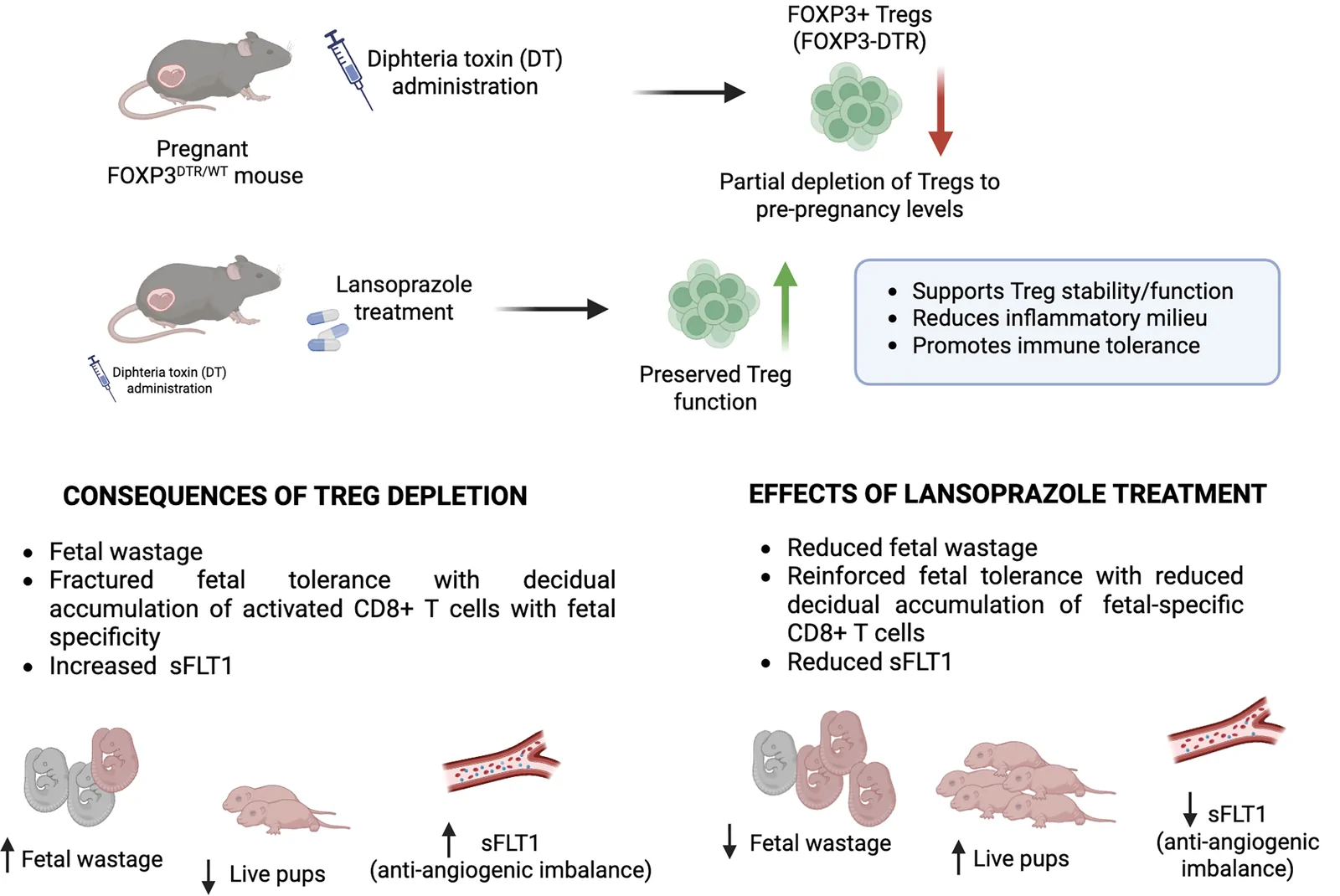
Proposed immunological mechanism underlying the beneficial effects of lansoprazole in a Treg-depletion model of preeclampsia Pregnant FOXP3^DTR/WT^ mice were administered diphtheria toxin (DT) to induce partial depletion of maternal Treg cells to pre-pregnancy levels, resulting in impaired maternal-fetal immune tolerance. Treg cell depletion promotes activation and accumulation of CD8 T cells in the decidua and secondary lymphoid organs, associated with increased fetal wastage, reduced numbers of live pups, and elevated circulating sFLT1 levels, consistent with an anti-angiogenic state. Lansoprazole treatment is proposed to restore maternal immune tolerance, reducing CD8 T cell activation and cytotoxicity, and attenuating the inflammatory milieu. These immunological effects are associated with reduced fetal wastage, increased numbers of live pups, and decreased sFLT1 levels.

These findings suggest that lansoprazole enhances maternal immune tolerance, potentially by supporting Treg function or counteracting the pro-inflammatory environment associated with their depletion. Overall, the mouse model highlights the interplay between immune tolerance mechanisms, vascular dysregulation, and preeclampsia pathology. Efficacy of lansoprazole in this system provides significant evidence of its therapeutic potential, making it a compelling candidate for further investigation in preclinical and clinical settings aimed at addressing the multifaceted challenges of preeclampsia.

An additional consideration relates to the dosing regimen used in the murine experiments. The dose of lansoprazole required to achieve therapeutic effects in our model is higher than doses typically used in clinical practice, and the intraperitoneal route of administration employed in mice differs substantially from the oral route used in humans. Consequently, drug exposure and pharmacokinetic profiles are not directly comparable between the experimental setting and clinical use. Therefore, our findings should not be interpreted as evidence that the same dosing regimen could be directly translated to patients. Future pharmacokinetic, dose-optimization, and efficacy studies using clinically relevant routes of administration will be required to determine whether comparable therapeutic effects can be achieved at exposures relevant to human pregnancy.

We would also like to acknowledge recent evidence raising questions about the safety of certain proton pump inhibitors during pregnancy, as these findings are relevant to the translational potential of lansoprazole as a candidate therapy for preeclampsia. A recent pharmacovigilance analysis of the FDA Adverse Event Reporting System (FAERS) by Dong et al. examined 1,099 pregnancy-related adverse-event reports listing proton pump inhibitors as the primary suspect drug across two decades (Q1 2004-Q1 2025) using reporting odds ratio (ROR) and Bayesian Confidence Propagation Neural Network (BCPNN) disproportionality methods. The authors detected signals for premature labor, low birth weight, fetal growth restriction, pre-eclampsia, and hemorrhagic complications, but importantly concluded that these signals were drug-specific rather than class-wide: pre-eclampsia and postpartum hemorrhage signals were largely confined to omeprazole (relative odds ratio [ROR] 1.95 and 3.14, respectively), preterm labor and low-birth-weight signals to pantoprazole, while lansoprazole—the candidate identified by our computational repurposing pipeline—showed no disproportionality for pre-eclampsia, fetal growth restriction, or preterm birth. Dong et al. themselves explicitly frame these results as “exploratory and hypothesis-generating, warranting cautious interpretation and confirmation through rigorously designed epidemiological and clinical studies”. Beyond the limitations inherent to spontaneous reporting (incomplete denominators, reporting and notoriety bias, lack of clinical metadata), the FAERS signals are subject to confounding by indication, which we consider the dominant interpretive issue in this literature. Women who receive proton pump inhibitors during pregnancy are disproportionately affected by obesity, prior bariatric or gastric bypass surgery, pregestational and gestational diabetes (and associated gastroparesis), severe GERD, and hyperemesis gravidarum, each of which is itself an independent risk factor for pre-eclampsia, preterm birth, fetal growth restriction, and low birth weight. Breddels et al. documented nearly 2-fold higher obesity prevalence among proton pump inhibitor users (21.6% vs. 11.7%) in the Swedish national cohort and explicitly acknowledged that confounding by indication could not be excluded. The active-comparator analysis of Choi et al. is particularly informative in this regard: when proton pump inhibitor-exposed pregnancies were compared not to non-users but to H2-receptor antagonist users—i.e., women sharing the same indication for acid suppression—the apparent excess risk of pre-eclampsia disappeared (RR 1.07 for all pre-eclampsia; RR 1.03 for preterm pre-eclampsia). Consistent with this, Hastie et al. reported that proton pump inhibitor use after 28 weeks was associated with a substantially reduced risk of preterm pre-eclampsia (aOR 0.63) and EO pre-eclampsia (aOR 0.41), aligning with the preclinical rationale for repurposing PPIs in placental disease. Collectively, these observations highlight that observational pharmacoepidemiology, whether based on spontaneous reporting systems such as FAERS or on national prescription registries, cannot reliably separate a drug effect from the effect of its underlying indication in this population. This is precisely why mechanistic *in vivo* evidence—such as the preclinical fetal wastage model triggered by partial depletion of maternal FOXP3^+^ Treg cells presented here—and ultimately appropriately powered randomized controlled trials are essential to establish whether lansoprazole exerts a causal, beneficial effect in pre-eclampsia, as our computational and experimental data suggest.

### Limitations of the study

Our study has several limitations that warrant consideration. The use of publicly available transcriptomic datasets posed challenges due to the inherent heterogeneity among studies, including differences in patient populations, sample preparation methods, and analytical platforms. We addressed these issues by employing integration methods such as MINT and meta-analysis, which allowed us to pool data effectively and produce robust, reproducible findings. However, it remains possible that our results may not generalize to cohorts from diverse ethnic backgrounds, individuals with different comorbidities, or those undergoing varying treatments. Further validation in independent and diverse populations is essential to assess the broader applicability of our findings.

Another limitation relates to our preclinical model of induced fetal wastage, which only replicates some aspects of preeclampsia. Particularly partial transient depletion of maternal FOXP3^+^ Treg cells is used to replicate the dampened expansion of these maternal cells in preeclampsia.^57^^,^^58^^,^^59^^,^^60^^,^^61^^,^^62^^,^^63^ This triggers elevated sFLT1 and reduced VEGF levels similar to preeclampsia. However, since other hallmark clinical features of preeclampsia, including hypertension and proteinuria, were not evaluated, the applicability to preeclampsia in humans remains uncertain. Thus, further combining these preclinical observations with human data is critical for validating the translational relevance of our findings.

An additional limitation is the reliance on bulk placental transcriptomic datasets. Although several of the included studies employed standardized placental sampling protocols, typically collecting tissue from defined central villous regions and excluding areas of infarction or hemorrhage, bulk expression profiles represent an average signal across multiple cell populations and microenvironments. Consequently, region-specific immune and angiogenic programs cannot be resolved, and immune deconvolution analyses cannot distinguish between maternal and fetal cellular contributions. Therefore, the inferred immune cell signatures should be interpreted as reflecting the overall placental immune landscape rather than specific maternal or fetal immune compartments. Future studies integrating single-cell and spatial transcriptomic approaches will be necessary to resolve the cellular origin and spatial localization of these immune populations, identify the anatomical sources of disease-associated molecular signatures, and further refine candidate biomarkers and therapeutic targets.

Our findings should be interpreted within the realistic possibilities and limits of drug repurposing as a discovery strategy. Although the field’s canonical successes—sildenafil for erectile dysfunction and subsequently pulmonary arterial hypertension, thalidomide and its analogs for multiple myeloma and erythema nodosum leprosum, minoxidil for androgenetic alopecia, metformin in oncology trials—demonstrate that legacy molecules can yield transformative new indications, the broader experience is more sobering: a Cambridge analysis concluded that only ∼2% of compounds entering clinical trials are ultimately launched for a genuinely different indication, and many computationally nominated candidates fail to translate.^91^^,^^93^ At the same time, repurposed candidates show approximately 3-fold higher overall approval rates (∼30% vs. ∼10% for new chemical entities) and substantially higher per-phase success because existing human safety, pharmacokinetic, formulation, and manufacturing data sharply reduce attrition risk in early development. Repositioning is therefore best understood not as a guaranteed shortcut but as a strategy that meaningfully shifts the probability and timeline of clinical translation, while still requiring indication-specific mechanistic validation. Our work is structured around exactly that principle: the connectivity-map-based signature-reversal pipeline serves as a hypothesis generator, and the fetal wastage model triggered by partial depletion of maternal FOXP3^+^ Treg cells provides the indication-specific mechanistic validation that this class of approach has too often omitted. Within this landscape, lansoprazole is a particularly instructive candidate. Consistent with our deliberate decision not to claim the gastric H^+^/K^+^ ATPase as the pharmacological target for the anti-preeclamptic effect—and instead to anchor the rationale in the phenotypic reversal of the preeclamptic transcriptional signature—lansoprazole has already been the subject of multiple repositioning programs operating through off-target mechanisms entirely unrelated to acid suppression. These include anticancer activity through autophagy modulation and inhibition of macrophage migration inhibitory factor (MIF) tautomerase activity, antimycobacterial activity against *Mycobacterium tuberculosis* via intracellular sulfoxide reduction to the active metabolite lansoprazole sulfide,^94^ and anti-fibrotic activity in models of idiopathic pulmonary fibrosis. This pre-existing repositioning footprint reinforces the central premise of our pipeline—that the molecule engages biologically meaningful targets beyond the proton pump—and provides additional human safety and pharmacokinetic data across diverse populations that would support eventual indication-specific clinical evaluation in preeclampsia.

While these findings identify lansoprazole as a potentially interesting candidate for further investigation, they should not be interpreted as evidence supporting its use for the prevention or treatment of preeclampsia. The observed effects in our experimental model provide supporting proof-of-concept data that lansoprazole may modulate pathways relevant to placental dysfunction and sFLT1 production. However, since not all clinical features of preeclampsia are reproduced, additional validation in complementary preclinical models and ultimately appropriately designed clinical studies will be required to determine whether these findings have therapeutic relevance in human pregnancy. Despite these limitations, our study provides a strong foundation for future research. The preeclampsia gene signature and associated biomarkers we identified have significant potential for multiple applications. Immediate next steps include validating the signature in retrospective human cohorts to confirm its reliability across diverse populations. These biomarkers could also serve as inputs for machine learning models designed for diagnostic or prognostic purposes, potentially enabling earlier detection and better management of preeclampsia.

## Resource availability

### Lead contact

Requests for further information and resources should be directed to and will be fulfilled by the lead contact, Marina Sirota (marina.sirota@ucsf.edu).

### Materials availability

This study did not generate new unique reagents.

### Data and code availability

• Data: All datasets analyzed in this study are publicly available. Accession numbers are listed in the key resources table.
• Code: All original code used to perform the analyses reported in this paper is publicly available at GitHub: https://github.com/msirota/preeclampsia.
• Additional information: Any additional information required to reanalyze the data reported in this paper is available from the lead contact upon request.

## Acknowledgments

This study was in part supported by March of Dimes Prematurity Research Centers (UCSF, Stanford, Ohio Collaborative), European Union – NextGenerationEU: National Center for Gene Therapy and Drugs based on RNA Technology, CN3 – (code:CN00000041), the NIH-NIAID through grants R01AI184537, R01AI175431 (SSW), the Burroughs Wellcome Fund Investigator in the Pathogenesis of Infectious Disease Award (grant #1011031) (SSW). We thank the NIH Tetramer Core Facility (supported by NIH-NIAID contract 75N93020D00005) for providing H-2K^b^:OVA_257-264_ tetramer used for identifying fetal-OVA-specific CD8 T cells. All figures were created with BioRender.com.

## Author contributions

R.A. performed the bioinformatic analyses and led the manuscript preparation; G.P., T.S., and A.L.S.validated the findings using mouse models; O.Y. and B.Y. contributed to the clinical interpretation of the results; A.S.T. conducted network analyses and supported manuscript writing; T.S., A.L.S., J.E., R.J.W., F.V., P.F.G., M.H.C., B.G., and T.T.O. reviewed the manuscript and provided valuable insights; M.S., S.S.W., and D.K.S. conceptualized and designed the study. All authors contributed to the final review and approval of the manuscript. Authorship order was determined through mutual agreement, prioritizing the chronological sequence of their contributions, with R.A. leading the bioinformatic analyses and manuscript preparation, and G.P. leading the experimental validation using mouse models.

## Declaration of interests

Dr. Federico Gherardini holds stock in HepaTx, Teiko.bio, and Cellanome. Dr. Ophelia Yin is funded for an investigator-led pilot trial by Thermo Fisher. Several co-authors are listed on the following patents: WO2020191297A1.

## STAR★Methods

### Key resources table

**Table:** 

REAGENT or RESOURCE	SOURCE	IDENTIFIER
**Antibodies**		

PE/Cy5 anti-mouse/human CD11b Antibody, Clone M1/70	BioLegend	Cat# 101210 RRID: AB_312793
PE/Cy5 anti-mouse/human CD45R/B220 Antibody, Clone RA3-B62	BioLegend	Cat# 103210 RRID: AB_312995
Alexa Fluor 700 anti-mouse/human CD44 Antibody, Clone IM7	BioLegend	Cat# 103026 RRID: AB_493713
eFluor 450 anti-mouse CD4 Antibody, Clone GK1.5	Invitrogen	Cat# 48-0041-82 RRID: AB_10718983
APC-eFluor 780 anti-mouse CD8alpha Antibody, Clone 536.7	Invitrogen	Cat# 47-0081-82 RRID: AB_1272185
PE/Cy5 anti-mouse CD11c Antibody, Clone N418	Invitrogen	Cat# 15-0114-82 RRID: AB_468717
PE/Cy5 anti-mouse F4/80 Antibody, Clone BM8	Invitrogen	Cat# 15-4801-82 RRID: AB_468798
FITC anti-mouse CD4 Antibody, Clone GK1.5	Invitrogen	Cat# 11-0041-85 RRID: AB_464893

**Chemicals, peptides, and recombinant proteins**		

H-2K^b^:OVA_257-64_ tetramer	NIH Tetramer Core	N/A
Diphtheria Toxin (DT)	Sigma	Cat# D0564
Lansoprazole	Sigma	Cat# L8533
Lansoprazole	Kroger (over the counter)	15 mg capsules
LIVE/DEAD Near-IR staining kit	ThermoFisher	L34976

**Critical Commercial Assays**		

Mouse sVEGFR1/Flt-1 DuoSet ELISA	R&D system	Cat# DY471
Mouse C-Reactive Protein DuoSet ELISA	R&D system	Cat# DY1829
Mouse VEGF DuoSet ELISA	R&D system	Cat# DY493

**Deposited data**		

Raw and analyzed data	NCBI GEO	GSE25906, GSE74341, GSE75010, GSE54618, GSE114691, GSE218039, GSE149437

**Experimental Models: Organisms/Strains**		

C57BL/6	The Jackson Laboratory	Strain Code: 000664
FOXP3^DTR/WT^	Breeding, Cincinnati Children’s Hospital	F1 female offpsirng from mating FOXP3^DTR^ hemi males, Kim et al.^65^ with C57BL/6 females
Balb/c	The Jackson Laboratory	Strain Code: 000651
Balb/c-Act-OVA-2W1S	Original gift from Marc Jenkins (University of Minnesota), with breeding at Cincinnati Children’s Hospital	Severance et al.^71^ Rowe et al.^66^

**Software and algorithms**		

R 4.4.2	R Foundation for Statistical Computing	https://cran.r-project.org/
MetaIntegrator 2.1.3	Haynes et al.^34^	https://cran.r-project.org/src/contrib/Archive/MetaIntegrator/
limma 3.62.1	Ritchie et al.^95^	https://bioconductor.org/packages/release/bioc/html/limma.html
GSVA 2.0.5	Hänzelmann et al.^36^	https://www.bioconductor.org/packages/release/bioc/html/GSVA.html
FlowJo	BD Biosciences	Version 10.7.1
GraphPad Prism	GraphPad Software	Version 10.1.1
Github repository		https://github.com/msirota/preeclampsia

### Experimental model and study participant details

#### Animals

C57BL/6 (H-2^b^) and Balb/c (H-2^d^) mice were purchased from the Jackson Laboratory. FOXP3^DTR/WT^ mice where half the maternal FOXP3^+^ cells are susceptible to DT-induced depletion have been described.^57^^,^^64^^,^^96^ OVA-2W1S transgenic mice were maintained on the Balb/c background after backcrossing for >10 generations, and the breeding strategy using OVA-2W1S males to sire allogeneic pregnancy in non-transgenic females has been described.^57^^,^^97^ For mating to establish allogeneic pregnancy, female mice of each strain were co-housed with males beginning 6–8 weeks of age. For maternal FOXP3^+^ cell depletion, FOXP3^DTR/WT^ or FOXP3^WT/WT^ control mice were administered purified DT daily (Sigma-Aldrich, USA) (0.5 μg first dose [E11.5]), followed by 0.1 μg each day thereafter for 2 additional days. Lansoprazole (4 mg/mL) was dissolved in sterile saline supplemented with 5% DMSO, and 150 μL (0.6 mg) was administered E10.5 to some mice one day prior to initiating DT treatment. Mice were randomly assigned to experimental groups. No blinding was performed since the experimental observations presented would be consistent irrespective of blinding and therefore blinding was not relevant in this study. The data presented in Figure 4 are representative of two independent experiments, and the data presented in Figure 5 are representative of four independent experiments. Individual data points represent individual mice. All experiments were performed under Cincinnati Children’s Hospital IACUC approved protocols (IACUC2023-1062).

#### Sex as a biological variable

This study used a preclinical mouse model of preeclampsia, focusing exclusively on female mice, since pregnancy is inherently specific to this sex. The experimental design leveraged transgenic female FOXP3^DTR/WT^ mice, which allowed for partial depletion of maternal Treg cells during pregnancy through DT administration. This depletion recapitulated hallmark features of human preeclampsia, including increased circulating sFLT1 levels, fetal wastage, and decidual infiltration of activated CD8 T cells. Male mice were used for breeding to sire allogeneic pregnancies, with particular use of male mice engineered to constitutively express model antigens (e.g., OVA) transforming these into surrogate fetal antigens. Given the focus on pregnancy-specific mechanisms and maternal immune adaptations, male mice were not used beyond their role in breeding. The findings are directly relevant to female physiology during pregnancy, as pregnacy and preeclampsia exclusively affects females. This sex-specific approach allowed us to faithfully model and study the pathophysiology of preeclampsia while testing the therapeutic potential of lansoprazole in mitigating pregnancy complications.

### Method details

#### Data

We curated six independent datasets from the Gene Expression Omnibus (GEO) repository, each profiling placental tissue from pregnant individuals under varying clinical conditions (Table 1) totaling 151 cases and 96 controls. To ensure a focused and biologically meaningful analysis, we selected samples representing pregnancies affected by EO preeclampsia (GA ≤34 weeks) as cases. For the control group, we chose samples from pregnancies resulting in preterm births (GA <37 weeks), matching for GA to minimize confounding effects. This stringent selection criteria enabled us to isolate transcriptomics differences attributable specifically to EO preeclampsia, enhancing the robustness and interpretability of our findings.

To further extend our analysis and enable cross-tissue comparisons, we incorporated a longitudinal transcriptomics dataset profiling blood samples from pregnant individuals over time (13 cases and 71 controls) (Table 2). This dataset captures dynamic changes in gene expression throughout gestation, offering a unique opportunity to investigate temporal patterns associated with preeclampsia. By leveraging longitudinal samples, we aim to uncover temporal biomarkers and track molecular shifts, providing valuable insights into the systemic manifestations of the disease and aiding in the discovery of potential biomarkers for early diagnosis and monitoring.

Available clinical and demographic characteristics of the included cohorts were retrieved from the original studies whenever possible and are provided in Tables S7, S8, S9, S10, S11, S12, and S13.

#### Data preprocessing and integration

To facilitate the integration of transcriptomic datasets generated using different platforms, platform-specific preprocessing and normalization procedures were applied. RNA-sequencing datasets were reprocessed from raw sequencing data using the nf-core/rnaseq Nextflow pipeline, and gene expression values were subsequently converted to transcripts per million (TPM). For microarray studies, processed expression matrices were downloaded from the Gene Expression Omnibus (GEO). When necessary, microarray datasets were quantile normalized to ensure comparability of expression distributions across samples. All datasets were then mapped to a common gene identifier space and analyzed using study-level effect sizes within the meta-analysis framework, thereby minimizing the impact of platform-specific differences.

Clinical metadata were variably reported across the included public datasets. Available clinical and demographic characteristics were retrieved from the original studies whenever possible and are provided in Tables S7, S8, S9, S10, S11, S12, and S13. Due to the incomplete and heterogeneous reporting of clinical covariates across studies, these variables could not be systematically incorporated into the analyses. Instead, datasets were integrated using a meta-analysis framework based on study-level effect sizes, which explicitly accounts for between-study heterogeneity.

#### MetaIntegrator

For the meta-analysis of gene expression datasets, we employed the MetaIntegrator framework,^34^ an established computational method specifically designed to integrate and analyze transcriptomic data from multiple independent studies. Meta-analysis of gene expression data is crucial in consolidating findings across different datasets, enabling the identification of robust and reproducible gene signatures associated with the condition of interest, even in the presence of biological and technical variability.

MetaIntegrator calculates effect sizes for each gene across all datasets, using the standardized mean differences (SMDs) to quantify the magnitude and direction of association between gene expression and disease state:

$SMD_{between}=\frac{\underline{x_{1}}-\underline{x_{2}}}{s_{pooled}}$, with

$$s_{pooled}=\sqrt{\frac{\left(n_{1}-1\right)s_{1}^{2}+\left(n_{2}-1\right)s_{2}^{2}}{\left(n_{1}-1\right)+\left(n_{2}-1\right)}}$$

where ${\underline{x}}_{1}$ is the mean of group 1, ${\underline{x}}_{2}$ is the mean of group 2, *n*_1_ is the number of observations in group 1, *n*_2_ is the number of observations in group 2, $s_{1}^{2}$ is the variance of group 1 and $s_{2}^{2}$ is the variance of group 2. *s*_*pooled*_ is therefore the pooled standard deviation, which is a weighted average of the standard deviations of the two groups.

To address inter-study heterogeneity - a common issue in meta-analysis - the method uses a random-effects model. This statistical approach assumes that effect sizes may vary across studies, accounting for both within- and between-study variability. The random-effects model thus provides a more generalizable estimate of effect size, reducing the risk of bias that might arise from directly pooling data.

Gene signatures are then identified based on combined effect sizes and significance thresholds, prioritizing genes with consistent associations across studies. This rigorous approach provides a robust and reproducible set of candidate genes for further validation and exploration, particularly valuable for complex diseases where single-cohort studies may lack sufficient power.

#### MINT

We employed the MINT (Multivariate INTegrative) method to integrate gene expression data across multiple cohorts, leveraging the multiblock framework within the mixOmics R package. MINT is specifically designed to handle multiple datasets, or “blocks”, which represent data from independent studies. This method enables us to identify robust gene signatures associated with a phenotype of interest across heterogeneous datasets by maximizing the covariance between the predictors (gene expression) and the response variable (e.g., disease status), while accounting for variations between studies.

MINT operates by optimizing a multivariate model for each block, where the data matrix *X*^(*k*)^ for the *k*-th study (block) contains observations (e.g., samples) as rows and features (e.g., gene expression levels) as columns. To capture both inter-study and intra-study variations, MINT decomposes each block-specific data matrix into two parts: a common component that captures shared patterns across studies, and a residual component unique to each block. The method seeks to maximize the covariance between the common components of the predictors and the response variable using a sparse partial least squares (sPLS) approach, which selects only the most relevant features to avoid overfitting and to enhance biological interpretability.

Formally, given *K* blocks, MINT maximizes the following criterion:

$$\sum_{k=1}^{K}\;Cov{\left(X^{\left(k\right)}\cdot \;\omega^{\left(k\right)},Y\right)}^{2}\text{,}$$

Where *ω*^(*k*)^ is the loading vector for the *k*-th block, and *Y* is the response matrix. The method iteratively estimates *ω*^(*k*)^ for each block, balancing both within-block relevance and across-block consistency.

By integrating multiple datasets in this way, MINT provides a unified model that can generalize well across different cohorts. The resulting signature thus reflects features that consistently correlate with the disease or phenotype across all studies, minimizing the influence of cohort-specific biases. This integrative approach is particularly beneficial for multi-cohort studies, as it enables the identification of biomarkers with cross-cohort stability and potential clinical relevance.

#### Longitudinal analysis

We applied a linear mixed-effects modeling approach to analyze longitudinal blood gene expression data from preeclamptic patients and controls, focusing on genes identified in the MINT signature from placental tissue. Using the lme4 package in R, we constructed a model tailored to capture both the fixed effects of disease class and gestational age (GA), as well as the random effects inherent to repeated measures within individuals. The model was defined as:

exprValue∼class∗poly(gaBloodDraw,degree=2)+(1|patientid)

The fixed effects included the disease *class* (control or preeclampsia) and its interaction with a second-degree polynomial of gestational age at the time of blood draw (*gaBloodDraw*). This allowed us to model complex, non-linear trajectories of gene expression over the course of pregnancy, providing detailed insights into how these changes diverge between preeclamptic and healthy pregnancies. The inclusion of interaction terms was crucial for capturing the dynamic interplay between disease status and temporal gene expression patterns. To account for within-subject variability, we included a random intercept for *patientid*, addressing the correlation between repeated measures from the same individual. This approach enabled us to isolate individual-specific baseline expression levels, enhancing the precision of our estimates for the population-level effects. By integrating this rigorous mixed-effects framework, we successfully leveraged longitudinal data to uncover temporal gene expression dynamics associated with preeclampsia. Our analysis provides a robust link between tissue-derived MINT signatures and their manifestation in blood, advancing our understanding of systemic molecular disruptions during pregnancy and offering a foundation for future investigations into predictive biomarkers or therapeutic targets.

#### Cell-mixture deconvolution

Cell-mixture deconvolution is a computational approach designed to infer the proportions of distinct cell types present within a bulk tissue sample. This method enables the dissection of complex biological samples into their cellular components, offering a deeper understanding of the sample’s cellular heterogeneity. For this analysis, we utilized the ImmunoStates basis matrix,^44^ a reference framework comprising gene expression profiles for well-characterized immune cell types, including T cells, B cells, monocytes, and NK cells, among others. The deconvolution process leverages a set of marker genes that are selectively expressed by specific cell types. By comparing the expression levels of these markers in the sample to those in the reference basis matrix, a linear modeling framework is applied to estimate the relative proportions of each cell type. This quantitative inference is crucial for studying immune-related diseases and for understanding how shifts in cellular composition may reflect pathological states or therapeutic responses. Importantly, this method enables the analysis of bulk RNA-sequencing data, which represents the averaged expression across all cells in a sample, and resolves it into meaningful contributions from individual cell types. By using the ImmunoStates matrix, we tailored our analysis to focus on immune cells, offering insights into the immune landscape of the disease. This approach not only enhances our ability to interpret gene expression data but also provides a critical foundation for identifying potential biomarkers, exploring disease mechanisms, and developing targeted therapies.

#### Computational drug repurposing pipeline

Inspired by the Kolmogorov-Smirnov statistic and the framework introduced by Lamb et al.^98^ in the original CMap database, the pipeline employs a nonparametric rank-based method to evaluate the concordance between disease-associated gene expression profiles and drug-induced expression signatures. A prefiltering step, adapted from prior drug repurposing methodologies, was applied to refine the input data. Genome-wide differential expression profiles from the CMap database were then analyzed, and candidate drugs were ranked based on the statistical significance of their reverse correlation with the preeclampsia signature, with adjustments for multiple testing. For drugs tested under varying conditions, the profile with the strongest reversal was selected for each compound.

#### Network analysis

To better understand the potential relationship between the top drug and preeclampsia, we explored relationships between genes, compounds, and related diseases via the SPOKE knowledge network (55), which includes information from DrugBank, ChEMBL, and Gene Ontology. Shortest paths between lansoprazole (CHEMBL ID: 480) and pre-eclampsia (Disease Ontology ID: 10591) were identified and combined.

All shortest paths were filtered with inclusion of.

• Nodes: Compound, Pharmacologic Class, Gene, and Disease nodes (exclusion of Anatomy, SideEffect, Protein, miRNA, and CellularComponent nodes).
• Edges: Associates_DaG, Downregulates_CdG, Downregulates_GpdG, Downregulates_KGdG, Downregulates_OGdG, Hasrole_ChC, Includes_PCiC, Isa_DiD, Marker_Pos_GmpD, Treats_CtD, Upregulates_CuG, Upregulates_GpuG, Upregulates_KGuG edges (exclusion of Contraindicates_CcD, Affects_CamG, Resembles_DrD edges).
  ○ Edge attribute filters include Treats_CtD with at compound in clinical trial phase 3 or greater; Upregulates_KGuG, Downregulates_KGdG, and Presents_DpS with p-value <1e−4 based on statistical tests from edge-specific database sources.

Some more information of select edges.

• CdG or CuG: Compound downregulates/upregulates gene: according to transcriptional profiles from LINCS L1000
• GpdG or GpuG: Gene product downregulates/upregulates gene: according to transcriptional profiles from LINCS L1000
• KGdG or KGuG: Knockdown of gene downregulates/upregulates gene: knockdown or knockout (using short hairpin RNA or CRISPR) of one gene affects another according to transcriptional profiles from LINCS L1000
• OGdG or OGuG: Overexpression of gene downregulates/upregulates gene: according to transcriptional profiles from LINCS L1000
• CtD: Compound treats disease. Sources include ChEMBL, DrugCentral, and DrugMatrix.

More details can be found from the SPOKE publication (55).

#### Lansoprazole treatment

The lansoprazole dose used in this study was selected based on our previous work evaluating its effects in a murine model of LPS-induced inflammation and fetal wastage^14^ and is consistent with doses used in other preclinical studies investigating its anti-tumor, neuroprotective, and cytoprotective properties.^99^^,^^100^^,^^101^ Although lansoprazole is primarily used as a proton pump inhibitor, accumulating evidence indicates that it exerts biological effects beyond gastric acid suppression. Notably, lansoprazole has been shown to induce the stress-response enzyme heme oxygenase-1 (HO-1),^102^ a pathway of particular relevance to preeclampsia. HO-1 negatively regulates sFlt-1 expression, and HO-1 deficiency has been associated with the development of a preeclampsia-like phenotype in mice.^103^ Furthermore, pharmacological induction of HO-1 has been shown to reduce circulating sFlt-1 levels and improve disease-related features in preclinical models.^104^ Therefore, the beneficial effects of lansoprazole observed in our study may reflect not only its established pharmacological activity but also systemic antioxidative and cytoprotective mechanisms mediated, at least in part, through HO-1 induction.

#### ELISA

Levels of Flt1/sVEGFR1, C-reactive protein, and VEGF in mouse sera were quantified by ELISA using commercial kits (R&D Systems; DY471 [Flt1], DY1829 [CRP], and DY493 [VEGF]).

#### Tetramer staining and flow cytometry

Single cell splenocytes suspensions were prepared by gentle tissue dissociation. Decidua tissue was isolated and processed by removing individual placentas from fetuses with separation at the labyrinth and junctional zone interface as described.^70^ Tissue was placed in RBC lysis buffer and single-cell suspension generated by gentle grinding between frosted glass slides. Complete DMEM was used to quench the lysis reaction and samples were then filtered through 70 μm cell strainers and pelleted by centrifugation (530 g for 5 min). For tracking CD8 T cells with H-2K^b^:OVA_257-264_ specificity, single cell suspensions from each tissue were incubated with PE-conjugated or APC-conjugated tetramers, with further enrichment of splenocytes using anti-fluorochrome microbeads (Miltenyi Biotec). Cells were analyzed on a FACSCanto cytometer (BD Biosciences) and analyzed using FlowJo (TreeStar) software.

### Quantification and statistical analysis

We employed a comprehensive suite of statistical methods to ensure robust and reliable analysis across our multi-cohort and multidimensional datasets. For the meta-analysis of gene expression data, pathways and cell types, we utilized the MetaIntegrator framework, which calculates standardized mean differences and applies a random-effects model to account for inter-study variability. To control for multiple hypothesis testing and reduce the likelihood of false positives, the framework implements FDR correction, thereby identifying significant genes, pathways or cell types associated with early-onset preeclampsia with greater confidence.

Cell mixture deconvolution was performed using a linear modeling approach, leveraging the ImmunoStates basis matrix and marker gene expression to estimate immune cell proportions within bulk RNA-sequencing samples.

For the analysis of longitudinal transcriptomic data, we applied a linear mixed-effects model with fixed effects for disease class and GA (modeled as a second-degree polynomial), and random intercepts for individual patients to account for intra-subject variability due to repeated measures.

In our computational drug repurposing pipeline, we employed a nonparametric rank-based approach inspired by the Kolmogorov-Smirnov statistic to assess the concordance between disease-associated and drug-induced gene expression profiles. Permutation analysis was carried out to evaluate the statistical significance of drug-disease profile concordance, and drug candidates with q-values <0.05 and reversal scores <0 (indicating signature reversal) were prioritized for further investigation.

Network analysis was conducted using the SPOKE knowledge network, where shortest paths between preeclampsia and candidate drugs were evaluated. Statistically significant edges (e.g., *p*-value <1e−4) were identified based on edge-specific database sources, providing insights into the relationships among genes, compounds, and diseases.

For experimental validation, female mice were randomly assigned to experimental or control groups. One-way ANOVA was used to analyze mean differences between two or more groups (Prism, GraphPad). For each analysis, a *p*-value of <0.05 was taken as statistical significance. Unless otherwise indicated, statistical significance is denoted in the figures as follows: ∗*p* < 0.05, ∗∗*p* < 0.01, ∗∗∗*p* < 0.001, and ∗∗∗∗*p* < 0.0001. The statistical tests used, and other relevant statistical details are reported in the corresponding figure legends. Other statistical analyses were conducted using appropriate software packages in R, ensuring reproducibility and transparency in our approach.

#### Study approval

The study is approved under Cincinnati Children’s Hospital IACUC approved protocols (IACUC2023-1062).

## Supplemental information

Document S1. Figures S1–S6

Table S1. Upregulated and downregulated genes identified by meta-analysis of six placental preeclampsia datasets

Table S2. Differentially active pathways identified by meta-analysis of early-onset preeclampsia placental datasets

Table S3. Differentially abundant cell types identified by meta-analysis of early-onset preeclampsia placental datasets

Table S4. Candidate drugs predicted to reverse the early-onset preeclampsia transcriptional signature using Connectivity Map-based drug repurposing

Table S5. Differentially expressed genes defining the preterm birth-specific transcriptional signature

Table S6. Genes from the early-onset preeclampsia transcriptional signature represented in the Connectivity Map

Table S7. Clinical metadata for samples included in the GSE25906 dataset

Table S8. Clinical metadata for samples included in the GSE74341 dataset

Table S9. Clinical metadata for samples included in the GSE75010 dataset

Table S10. Clinical metadata for samples included in the GSE54618 dataset

Table S11. Clinical metadata for samples included in the GSE114691 dataset

Table S12. Clinical metadata for samples included in the GSE218039 dataset

Table S13. Clinical metadata for samples included in the GSE149437 dataset
