# Supplementary material for: Integrative transcriptomic analysis and preclinical validation identify lansoprazole as a therapeutic candidate for preeclampsia
Source: iScience. 2026 Oct 31;29(11):117963. doi: 10.1016/j.isci.2026.117963 (PMC13743939; doi:10.1016/j.isci.2026.117963)
Supplement: Supplementary file 1 [file mmc1.pdf]

iScience, Volume *29*

Supplemental information

Integrative transcriptomic analysis and preclinical validation identify lansoprazole as a therapeutic candidate for preeclampsia

Romina Appierdo, Giang Pham, Ophelia Yin, Alice S. Tang, Tzu-yu Shao, Ashley L. Severance, Bahar Yilmaz, Jakob Einhaus, Ronald J. Wong, Francesco Vallania, Pier Federico Gherardini, Manuela Helmer-Citterich, Brice Gaudillière, Tomiko T. Oskotsky, David K. Stevenson, Sing Sing Way, and Marina Sirota

A B

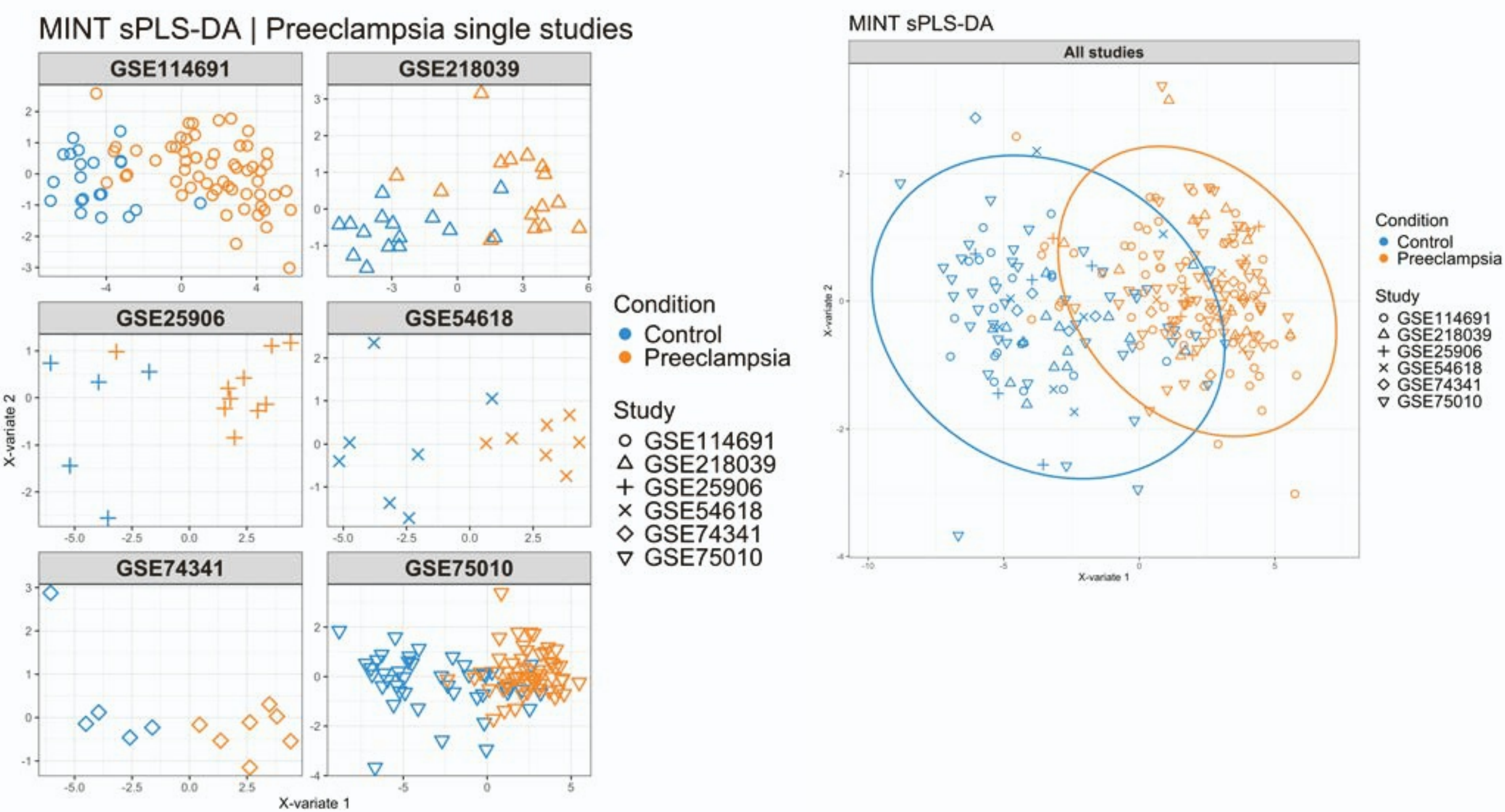

Supplementary Figure 1. Projection of latent structures (PLS) plots from the MINT sPLS-DA analysis for preeclampsia datasets.

Individual PLS plots for each of the six placental datasets (GSE74341, GSE75010, GSE25906, GSE114691, GSE218039, and GSE54618). The x- and y-axes represent the first two latent components, with each point representing a sample. Blue points indicate controls, and orange points indicate preeclampsia cases. MINT successfully distinguishes between preeclampsia and control groups within individual datasets, despite heterogeneity between studies (**A**). Combined projection of all six datasets after MINT integration. The x- and y-axes represent the first two common latent components derived from the integrated data. Each point represents a sample, color-coded by condition (blue for controls and orange for preeclampsia) and shaped by dataset. The ellipses represent the 95% confidence regions for each condition. This plot demonstrates successful integration of multi-study data, showing clear separation between preeclampsia and control samples, validating MINT's capability to adjust for study-specific biases while preserving disease-specific signals (**B**).

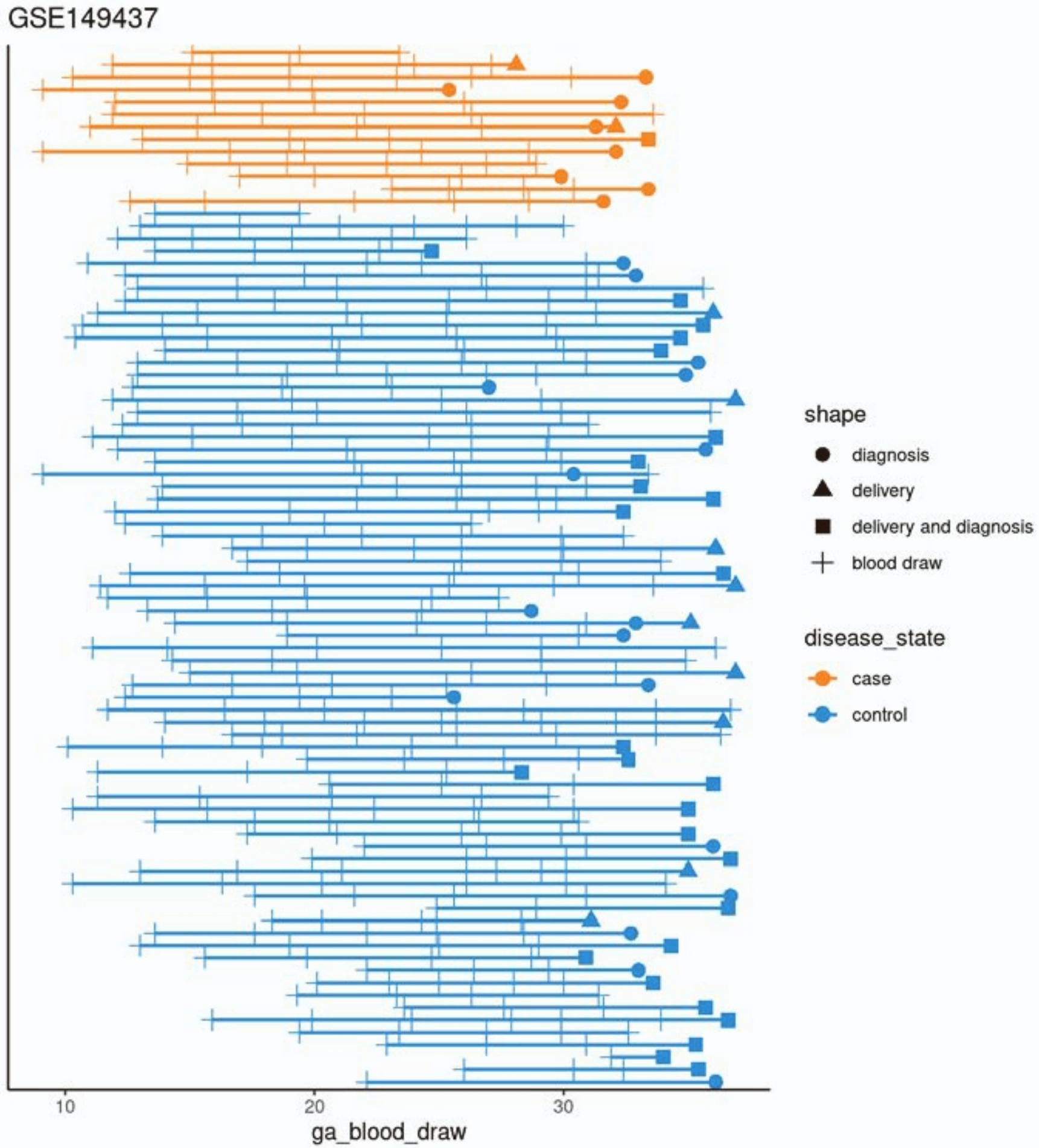

Supplementary Figure 2. Longitudinal blood dataset sampling strategy.
Longitudinal sampling timeline of blood draws for the GSE149437 dataset. The x-axis represents the gestational age (GA) at the time of each blood draw, while the y-axis shows individual participants. Each horizontal line corresponds to a participant, with symbols indicating key events: diagnosis (circles), delivery (triangles), and instances where both diagnosis and delivery occurred on the same day (squares). Blood draws are represented by tick marks along the lines. Orange lines represent preeclampsia cases, and blue lines represent controls. This longitudinal structure captures multiple sampling points per participant, allowing for the dynamic analysis of gene expression changes over time and comparison between preeclamptic and control pregnancies.

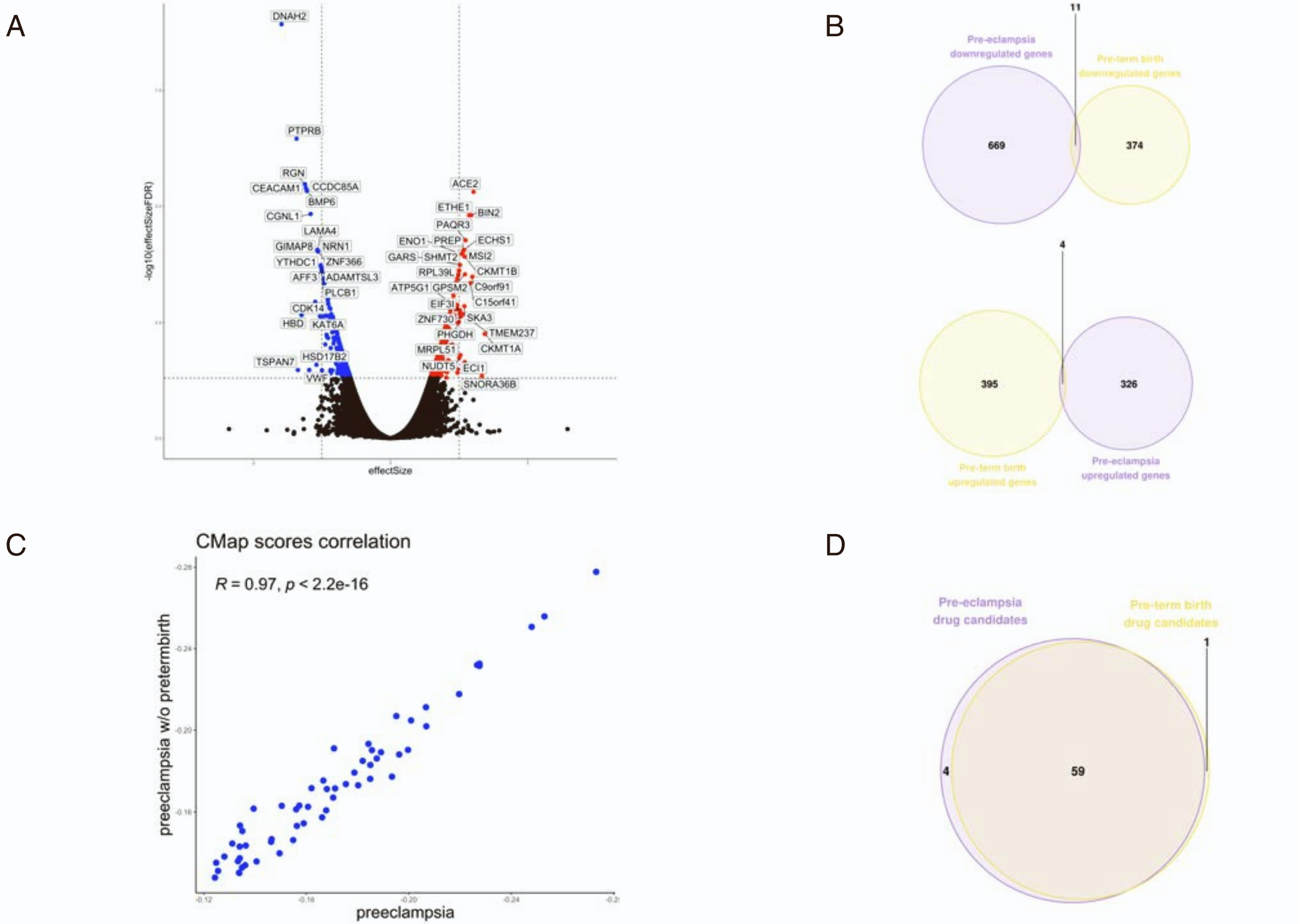

Supplementary Figure 3. Sensitivity analysis removing pre-term birth signal from preeclampsia signature. Volcano plot showing the results of meta-analysis for the preterm birth-specific signature. The x-axis represents the effect size, and the y-axis represents the -log10(FDR) of the meta-analysis. Differentially expressed genes (DEGs) are highlighted in blue (downregulated) and red (upregulated) (**A**). Venn diagrams comparing differentially expressed genes (DEGs) between the preeclampsia-specific signature and the preterm birth-specific signature. The top diagram shows overlap among downregulated genes, and the bottom diagram shows overlap among upregulated genes (**B**). Correlation between CMap scores of the drug candidates identified from the preeclampsia analysis before and after removing the preterm birth signature. The high correlation ($R = 0.97$, $p < 2.2e\text{-}16$) demonstrates that the drug predictions are nearly identical in both cases, confirming that the preeclampsia drug repurposing results are not influenced by signals associated with preterm birth (**C**). Venn diagram showing the overlap of drug candidates identified for preeclampsia before and after removing the preterm birth signature. Of the 63 drugs identified for preeclampsia, 59 overlapped with the candidates identified after adjusting for the preterm birth signal, indicating high consistency in the drug repurposing results and further validating the robustness of the preeclampsia-specific signature (**D**).

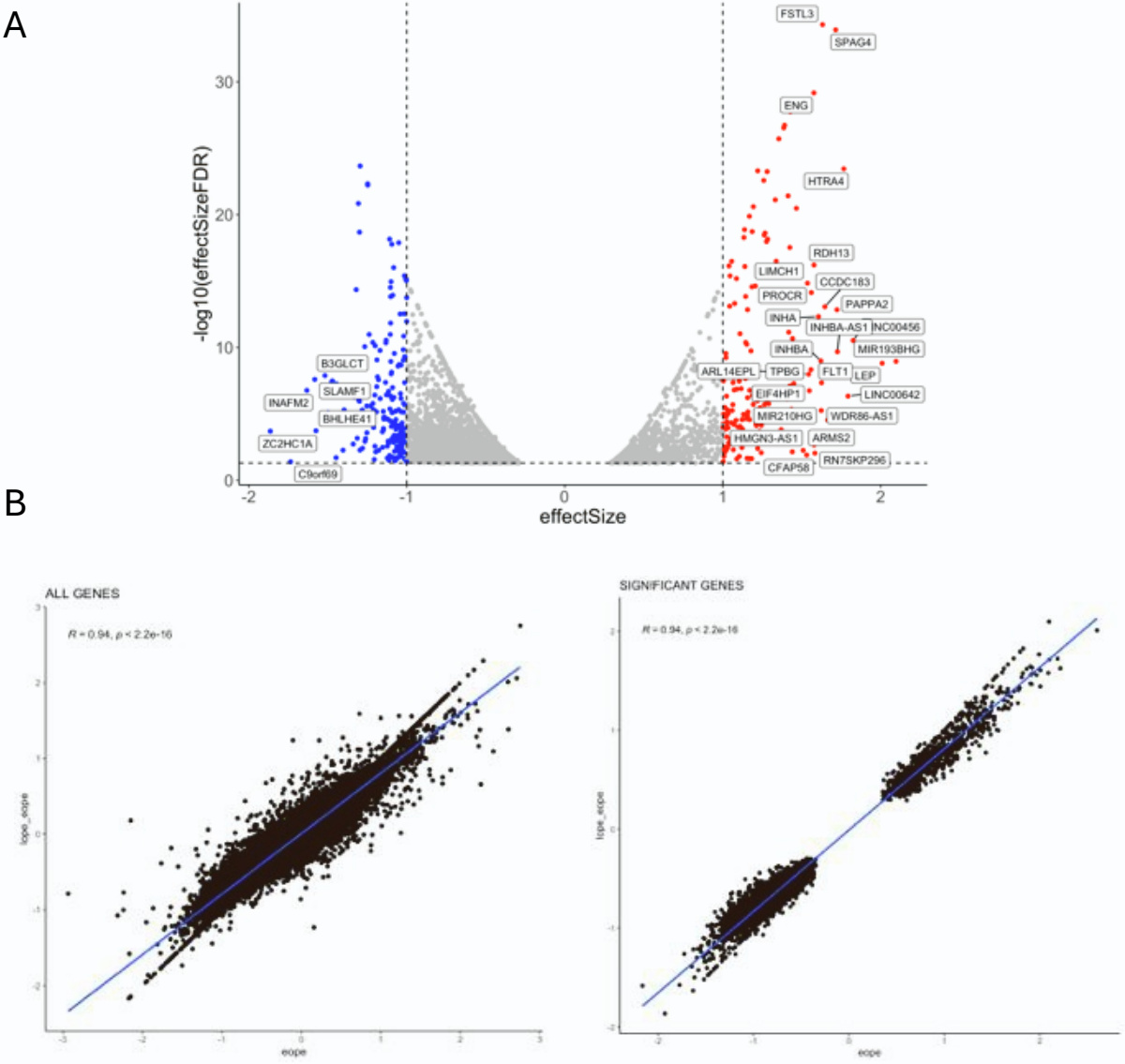

Supplementary Figure 4. Preeclampsia transcriptional signature and its consistency across early- and late-onset disease.
(A) Volcano plot showing differentially expressed genes. A total of 1,066 genes were upregulated and 2,379 downregulated at FDR < 0.05. Among these, genes with an absolute effect size > 1 are highlighted in red (upregulated) and blue (downregulated). Notably, genes such as *FLT1*, *FSTL3*, *LEP*, and *ENG* were strongly upregulated, consistent with prior findings in preeclampsia.(B) Log–log plots comparing effect sizes between the early-onset vs preterm birth analysis and the combined (early- and late-onset) analysis. A strong positive correlation was observed in both all genes (left) and significantly differentially expressed genes (right), with Spearman $\rho = 0.94$, $p < 0.05$ in both cases. These findings indicate that the molecular signature of preeclampsia is preserved across both early- and late-onset forms of the disease.

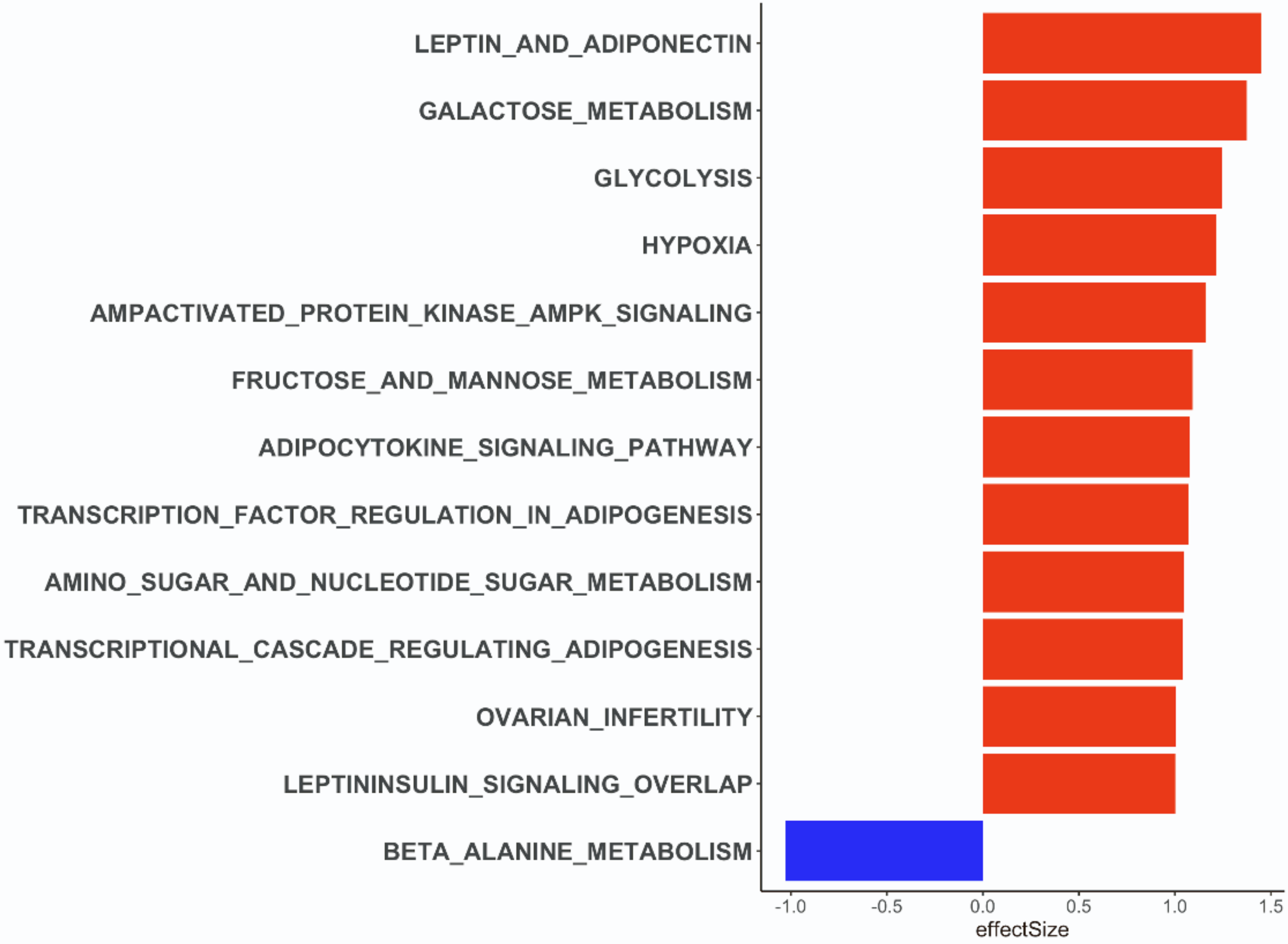

Supplementary Figure 5. Results of pathway activity score meta-analysis from the combined early- and late-onset preeclampsia datasets. The barplot shows pathways with significantly (FDR < 0.05) altered activity in preeclampsia based on effect sizes across studies (with abs(effect size) > 1). Pathways with positive effect sizes (red) are more active in preeclampsia, while those with negative effect sizes (blue) show reduced activity. Enriched pathways include those related to metabolism, hypoxia, adipogenesis, and ovarian function, highlighting the involvement of metabolic and endocrine dysregulation in the disease.

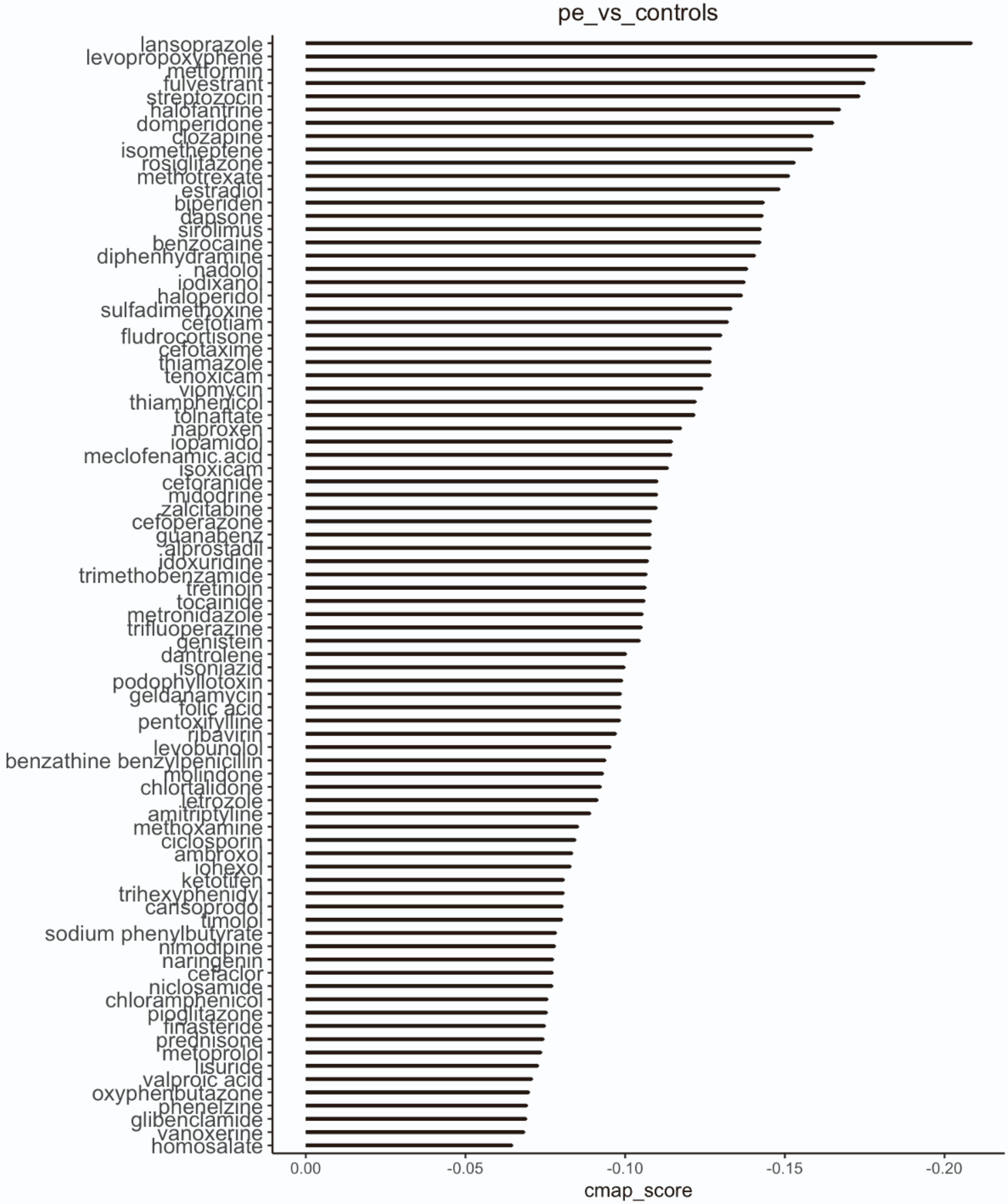

Supplementary Figure 6

Drug repurposing results based on the combined (early- and late- onset) preeclampsia gene expression signature. We identified 84 candidate compounds (FDR q < 0.05 and cMap score < 0) predicted to reverse the preeclampsia gene expression profile. The barplot shows the top negatively scored drugs ranked by cMap score. Lansoprazole emerged as the top hit, consistent with previous analyses, further supporting its potential for therapeutic repositioning in preeclampsia.
